# Morning or evening? Time of day does not affect body composition, cardiopulmonary performance, and muscle strength responses to combined training in women with obesity

**DOI:** 10.3389/fspor.2026.1914784

**Published:** 2026-09-15

**Authors:** Yan Leo Melo Vieira, Ana Vitoria Leca, Bruna Tabata Bernardes, Natalia Yumi Noronha, Carolina Batista Simoes, Pedro Henrique Reis Restier Pinheiro, Alicia Alvarenga Fernandes, Igor Jose Soares Rodrigues, Joao Miguel Guimaraes Flora, Lucas Tulio Lacerda, Diego Alcantara Borba, Lucas Rios Drummond, Michael Jackson Oliveira de Andrade, Julio Sergio Marchini, Erick P. de Oliveira, Dawit Albieiro Pinheiro Gonçalves, Camila Fernanda Cunha Brandao

**Affiliations:** 1 Department of Internal Medicine, Ribeirão Preto Medical School, São Paulo University, Ribeirão Preto, Brazil; 2 Multidisciplinary Center for Chronic Non-Communicable Diseases of the Central-West Region of Minas Gerais, State University of Minas Gerais, Divinópolis Campus, Minas Gerais, Brazil; 3 Postgraduate Program in Biosciences and Human Health, State University of Minas Gerais, Divinópolis, Minas Gerais, Brazil; 4 Department of Health Sciences, Ribeirão Preto Medical School, São Paulo University, Ribeirão Preto, Brazil; 5 Ergogenic & Systemic and Molecular Physiology Research Group (ERGOFIS), School of Physical Education, Physiotherapy and Occupational Therapy, Universidade Federal de Minas Gerais, Belo Horizonte, Brazil; 6 Sports Physiology Section (SFE), Sports Training Center (CTE), School of Physical Education, Physiotherapy and Occupational Therapy, Universidade Federal de Minas Gerais, Belo Horizonte, Brazil; 7 Laboratory of Nutrition, Exercise and Health (LaNES), School of Medicine, Federal University of Uberlândia, Minas Gerais, Brazil

**Keywords:** cardiopulmonary exercise testing, circadian rhythm, exercise, muscle strength, physical fitness

## Abstract

**Introduction:**

Obesity is characterized by adipocyte dysfunction, accompanied by impairments in oxidative metabolism and physical function. Although physical training is known to improve oxidative capacity, body composition, and functional performance, the magnitude of these adaptations may be influenced by several modulatory factors. Among these, circadian rhythm has emerged as a potential determinant of exercise-induced adaptations due to daily fluctuations in metabolic and physiological processes; however, its influence in women with obesity remains unclear. Therefore, this study aimed to investigate the effects of combined training performed at different times of the day (morning vs. evening) on body composition, cardiopulmonary performance, and muscle strength in previously untrained women with obesity.

**Methods:**

This randomized clinical trial (NCT06601660) included women with obesity (waist circumference ≥88 cm) aged 18–50 years. Participants were randomly assigned to a control group (CG; *n* = 13), a morning training group (MT; training performed between 07:00 and 10:00 a.m.; *n* = 17), or an evening training group (ET; training performed between 06:00 and 09:00 p.m.; *n* = 19). Assessments conducted before and after the intervention included body composition (InBody® 770), cardiopulmonary performance evaluated by cardiopulmonary exercise testing (CPET), muscle strength assessed by the one-repetition maximum (1RM) test, and cardiovascular parameters evaluated by blood pressure (BP) and heart rate (HR); the last three were performed at both a.m. and p.m. times. Only participants in the MT and ET groups underwent the intervention, which consisted of an 8-week combined training program performed three times per week. Each session included both resistance and aerobic exercise, with progressive increases in exercise intensity and duration throughout the intervention period. Participants assigned to the CG underwent assessments only.

**Results:**

Significant group-by-period interactions were observed for VO_2peak_ and bench-press and leg-press 1RM, whereas maximal treadmill velocity showed a significant period effect without a significant group-by-period interaction. Bench-press 1RM increased in MT during morning and evening testing by 5.79 and 5.31 kg, respectively, and in ET by 4.97 and 5.50 kg (all *p* < 0.001; *d* = 2.37–2.76), with gains in both training groups exceeding those observed in CG. Leg-press 1RM increased in MT by 22.20 and 29.01 kg and in ET by 18.40 and 17.11 kg during morning and evening testing, respectively (*p* ≤ 0.001; *d* = 1.13–1.92); however, only MT showed a greater overall change than CG. VO_2peak_ increased in MT during morning testing [2.42 mL·kg^−1^·min^−1^ (*p* = 0.004; *d* = 1.04)] and in ET during evening testing [1.57 mL·kg^−1^·min^−1^ (*p* = 0.048; *d* = 0.67)]. Maximal treadmill velocity and CPET duration improved over time, but their changes did not differ significantly among groups. Body-composition and cardiovascular adaptations were limited, with waist circumference increasing only in CG.

**Conclusion:**

Eight weeks of combined training performed either in the morning or evening improved maximal muscle strength and cardiopulmonary performance in women with obesity, while producing limited changes in body composition and cardiovascular measures. No consistent evidence of superior adaptations according to training time was observed, suggesting that both schedules can effectively promote health-related adaptations.

## 1 Introduction

Obesity is a chronic non-communicable disease characterized by excessive accumulation of adipose tissue and a heterogeneous clinical phenotype encompassing metabolic, inflammatory, and functional alterations beyond increased body mass (1). Despite substantial advances in understanding its underlying mechanisms and in developing therapeutic strategies, significant challenges to the prevention and management of obesity persist (2). In most cases, effective obesity management requires the establishment of a negative energy balance through a combination of reduced energy intake and increased energy expenditure; the latter is commonly achieved through regular physical exercise.

Physical training is an established non-pharmacological intervention for chronic diseases, including obesity and its associated comorbidities (3). Evidence suggests that combined training, defined as the integration of resistance and aerobic exercise within the same intervention, improves mitochondrial function (4, 5), reduces circulating sphingolipids and glycerophospholipids, as well as metabolites such as trimethylamine N-oxide, a recognized marker of cardiovascular risk (6, 7). Furthermore, combined training has been shown to modulate epigenetic mechanisms, including hypermethylation of genomic sites associated with transcriptional pathways involved in AMPK, TGF-β, and insulin signaling (8, 9). Beyond these molecular adaptations, chronic physical training promotes improvements in insulin sensitivity, glycemic control, plasma lipoprotein profiles, body composition, cardiopulmonary performance, and muscle strength (4–7, 10), thereby contributing to overall metabolic health and physical function (11).

Despite the well-established benefits of regular physical training *per se*, several studies have investigated whether performing exercise at different times of the day may elicit distinct training adaptations (12, 13). However, findings from individual studies remain inconsistent. Moholdt et al. (12) reported that 5 consecutive days of supervised physical training performed in the evening (06:30 p.m.) during an 11-day high-fat diet intervention elicited greater reductions in fasting blood glucose and larger improvements in insulin sensitivity than the same training performed in the morning (06:30 a.m.) in men with overweight or obesity. In contrast Teo et al. (15), demonstrated that 12-weeks of combined aerobic and resistance training performed either in the morning or in the evening resulted in comparable improvements in cardiopulmonary performance (VO_2peak_), body composition, and cardiometabolic outcomes, including in glycemic control [fasting and postprandial glucose and glycated hemoglobin (HbA1c)], insulin-related outcomes and insulin sensitivity; without consistent evidence that training time confers a superior benefit for cardiometabolic health.

Accordingly, a recent systematic review and meta-analysis (13, 14) found limited evidence supporting or refuting the hypothesis that performing exercise at a specific time of day results in greater improvements in performance- or health-related outcomes compared with other times of the day. Nevertheless, because most studies included in this meta-analysis were characterized by a high risk of bias, further investigations using rigorous methodological approaches, more diverse study populations, and adequate control and monitoring of potential confounding factors are warranted to determine whether exercise timing differentially influences the physiological and functional benefits of training.

Evidence from clinical studies involving individuals with overweight or obesity is similarly inconclusive. For example Moholdt et al. (12), reported comparable improvements in cardiopulmonary performance, assessed by VO_2peak_, following 5 consecutive days of supervised physical training performed either in the morning (06:30 a.m.) or in the evening (06:30 p.m.) during an 11-day high-fat diet intervention in men with overweight or obesity. However, only evening exercise reduced fasting glucose, fasting insulin, total cholesterol, triacylglycerol, and low-density lipoprotein (LDL) cholesterol concentrations, while also partially reversing high-fat diet-induced alterations in serum metabolomic profiles and lowering nocturnal glucose concentrations. In contrast Teo et al. (15), found no clear advantage of twelve-weeks of supervised multimodal physical training performed three times per week (60 min/session) either in the morning (08:00–10:00 a.m.) or in the evening (05:00–07:00 p.m.) with respect to glycemic outcomes in adults with overweight, including those with and without type 2 diabetes.

Each session consisted of 30 min of moderate-intensity treadmill walking (60%–70% VO_2peak_) followed by resistance training comprising four exercises (leg press, bench press, military press, and lat pulldown; three sets of 18, 15, and 12 repetitions at 45%, 50%, and 55% of 1RM, respectively). Although the intervention significantly improved HbA1c, fasting glucose, and postprandial glucose, these adaptations were similar between the morning and evening training groups. Collectively, these findings suggest that the effects of exercise timing on cardiometabolic markers, including fasting glucose, fasting insulin, HbA1c, postprandial glucose, blood lipid profile, and continuous glucose monitoring-derived outcomes, remain uncertain. Moreover, its potential influence on body composition and functional performance, particularly in women with obesity, has been insufficiently investigated.

This study aimed to investigate the effects of combined training performed at different times of the day, morning (07:00–10:00 a.m.) vs. evening (06:00–09:00 p.m.), on body composition, cardiopulmonary performance, muscle strength, and cardiovascular measures in previously untrained women with obesity, and to determine whether these adaptations were influenced by the time of day at which testing was performed. We hypothesized that ET would promote greater reductions in body fat mass, as well as greater improvements in cardiopulmonary performance and muscle strength, compared with morning training, particularly when outcomes were assessed in the evening.

## 2 Materials and methods

### 2.1 Study design and setting

This randomized clinical trial was approved by the Research Ethics Committee of the State University of Minas Gerais (UEMG), Divinópolis Unit, in accordance with the Brazilian National Health Council Resolution No. 466/2012 (CAAE: 59429722.1.0000.5115; approval number: 5.725.464), and was registered at ClinicalTrials.gov (NCT06601660). Data collection for participant characterization and cardiopulmonary assessments was conducted at the Research Laboratory on Metabolism, Physiology, and Physical Exercise. Muscle strength assessments were performed at the Research Laboratory on Strength Training for Health and Conditioning, while familiarization and intervention sessions took place in the institution's resistance training facility. During the initial contact with potential participants, all study procedures, as well as the associated risks and benefits, were explained via videoconference. Participants were enrolled only after completing two screening instruments, the Anamnesis Questionnaire and the Exercise Preparticipation Health Screening Questionnaire for Exercise Professionals (16, 17), and providing written informed consent.

### 2.2 Participants

The required sample size was estimated using G*Power software (version 3.1) based on an *F* test for repeated measures with a within–between interaction. The calculation assumed an effect size of 0.25, a statistical power of 80%, an alpha level of 0.05, three experimental groups, and two repeated measurements (pre- and post-intervention), resulting in a minimum sample size of 42 participants. This approach is based on the study design and planned statistical analyses rather than on a specific outcome variable. This study considered VO_2peak_ and 1RM as primary outcomes; anthropometry, body composition, and cardiovascular responses as secondary outcomes; and circadian preference as an exploratory characteristic of sample circadian typology. Participants were allocated to one of three experimental groups: morning training (MT), which performed combined training between 07:00 and 10:00 a.m.; evening training (ET), which performed combined training between 06:00 and 09:00 p.m.; and a control group (CG), which did not participate in the training intervention. Randomization was conducted using the RAND function in Microsoft Excel and stratified according to age and body mass index (BMI) to ensure balanced group allocation. Randomization was performed by YLMV under the supervision of CFCB and was not blinded. Due to the nature of the intervention, neither participants nor exercise supervisors could be blinded to group allocation. Investigators responsible for anthropometric, cardiopulmonary, and muscle strength assessments were not involved in the randomization process. However, allocation concealment was not maintained after group assignment, which is recognized as a limitation of the study. *A posteriori* power analysis was subsequently performed using G*Power 3.1 to determine whether the final sample retained adequate statistical power following participant attrition. Based on the observed effect size for upper-body maximal strength (1RM bench press; Cohen's *f* = 0.88), a total sample of 49 participants, three experimental groups, two repeated measurements, and an alpha level of 0.05, the achieved statistical power for the repeated-measures within–between interaction was 1.00 (100%). This indicates that the final sample exceeded the statistical power initially planned, and that participant losses did not compromise the ability of the study to detect the observed intervention effects.

The study was advertised through widely accessible media platforms, including newspapers, radio broadcasts, and Instagram. Subsequently, potential participants completed a Google Forms questionnaire comprising an anamnesis form and a health risk stratification assessment to determine eligibility. The inclusion criteria were as follows: women with obesity, defined by elevated body fat according to American College of Sports Medicine (ACSM) (17) criteria and a waist circumference ≥88 cm; absence of participation in structured physical training for at least three months; no use of anorexigenic or mood-stabilizing medications; no current nutritional counseling or dietary intervention; not engaged in night-shift work; no history of bariatric surgery; adequate health status to participate in physical training; and reproductive age between 18 and 50 years. The non-inclusion criteria comprised the presence of cardiovascular disease (except hypertension), joint or neuromuscular disorders, sleep–wake cycle disturbances, visual or neuropsychiatric impairments, and dependence on psychoactive substances. Participants were excluded from the study if they developed any disease during the intervention period, became pregnant or initiated lactation, or attended fewer than 80% of the prescribed training sessions.

Figure 1 shows that, during the recruitment phase, 705 responses were received through the online screening questionnaire. After removing duplicate submissions, 678 valid responses remained. Of these, 179 women met the initial eligibility criteria and were invited to participate in the study. A total of 133 participants completed the baseline assessments, including anthropometric, body composition, and physical performance evaluations, and were subsequently allocated to one of three groups: MT (*n* = 49), ET (*n* = 63), or CG (*n* = 21). Regarding the recruitment period, 51 participants completed the baseline, whereas an additional 82 participants were enrolled, resulting in a total of 133 participants assessed prior to the intervention. Before completing the intervention, 77 participants (57.9%) withdrew from the study, including 30 from the MT group (61.2%), 39 from the ET group (61.9%), and 8 from the CG (38.1%). The primary reason for withdrawal was the inability to attend the scheduled training sessions and assessment visits (7:00–10:00 a.m. or 6:00–9:00 p.m.) due to conflicts with work, family, or other personal commitments. Consequently, 56 participants (MT: *n* = 19, ET: *n* = 24, and CG: *n* = 13) completed the post-intervention assessments. After applying the predefined exclusion criteria (attendance <85%, participation in other exercise programs, or illness/injury), seven participants were excluded from the final analyses, resulting in a final sample of 49 participants (MT: *n* = 17, ET: *n* = 19, and CG: *n* = 13), all of whom attended at least 85% of the prescribed intervention sessions (≥20 of 24 sessions). Although these participants were excluded from the final statistical analyses, they were permitted to complete the post-intervention assessments to support their personal health monitoring and ensure appropriate ethical follow-up.

**Figure 1:**
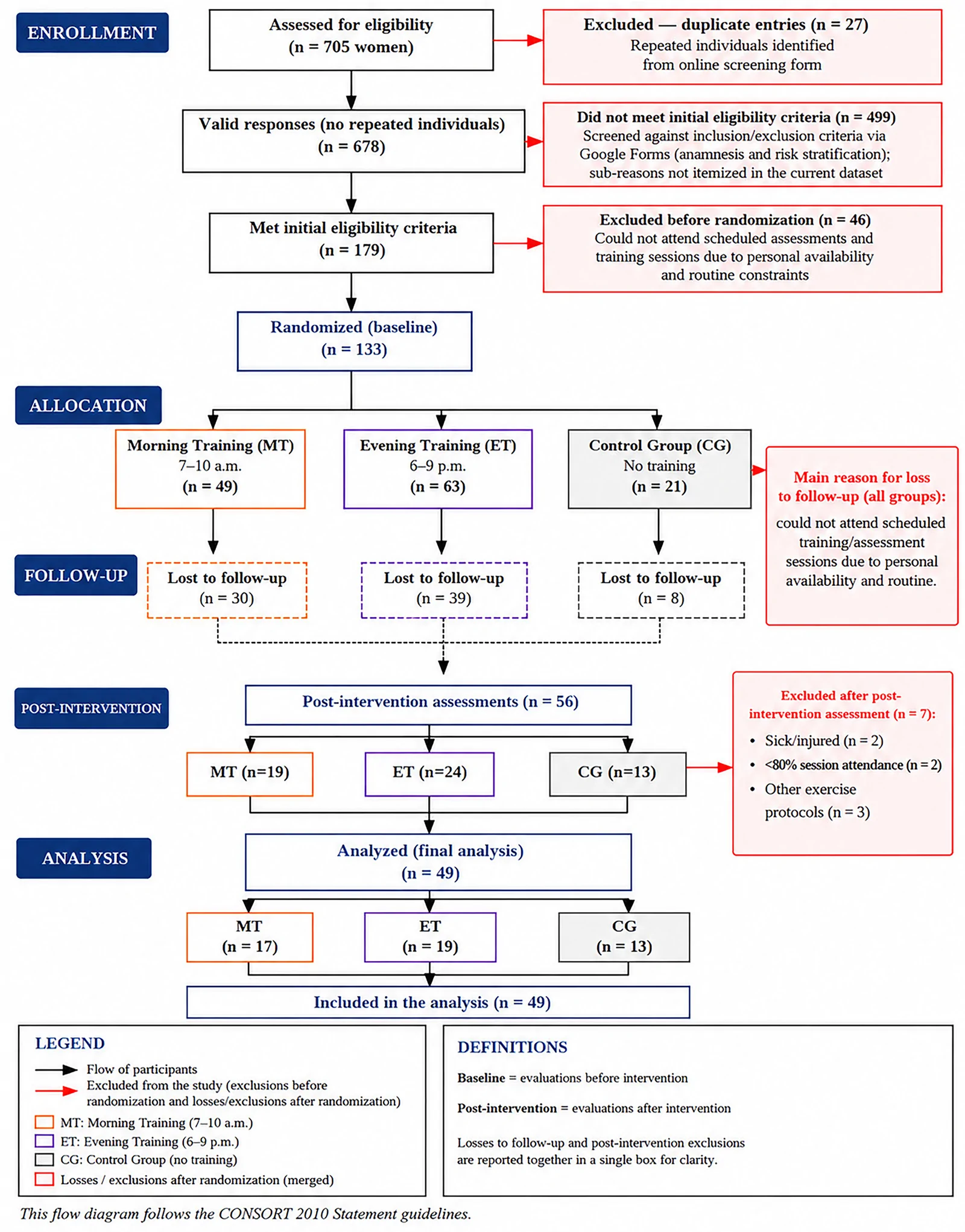
Sample size and participants’ distribution.

### 2.3 Experimental design

Figure 2 illustrates the overall study timeline, which spanned a total of 13 weeks. Following confirmation of eligibility, participants underwent three weeks of baseline assessments, including anthropometric and body composition measurements, familiarization with the training protocol, and physical performance testing; CPET, cardiovascular responses, and 1RM were assessed twice, with a 60 h interval between sessions: once in the morning (07:00–10:00) and once in the evening (18:00–21:00). These assessments were performed at both time points: pre- and post-intervention, for all three groups. Subsequently, participants completed an eight-week combined training intervention that was performed three times per week. Upon completion of the intervention, the same battery of assessments and physical tests was repeated over two weeks.

**Figure 2:**
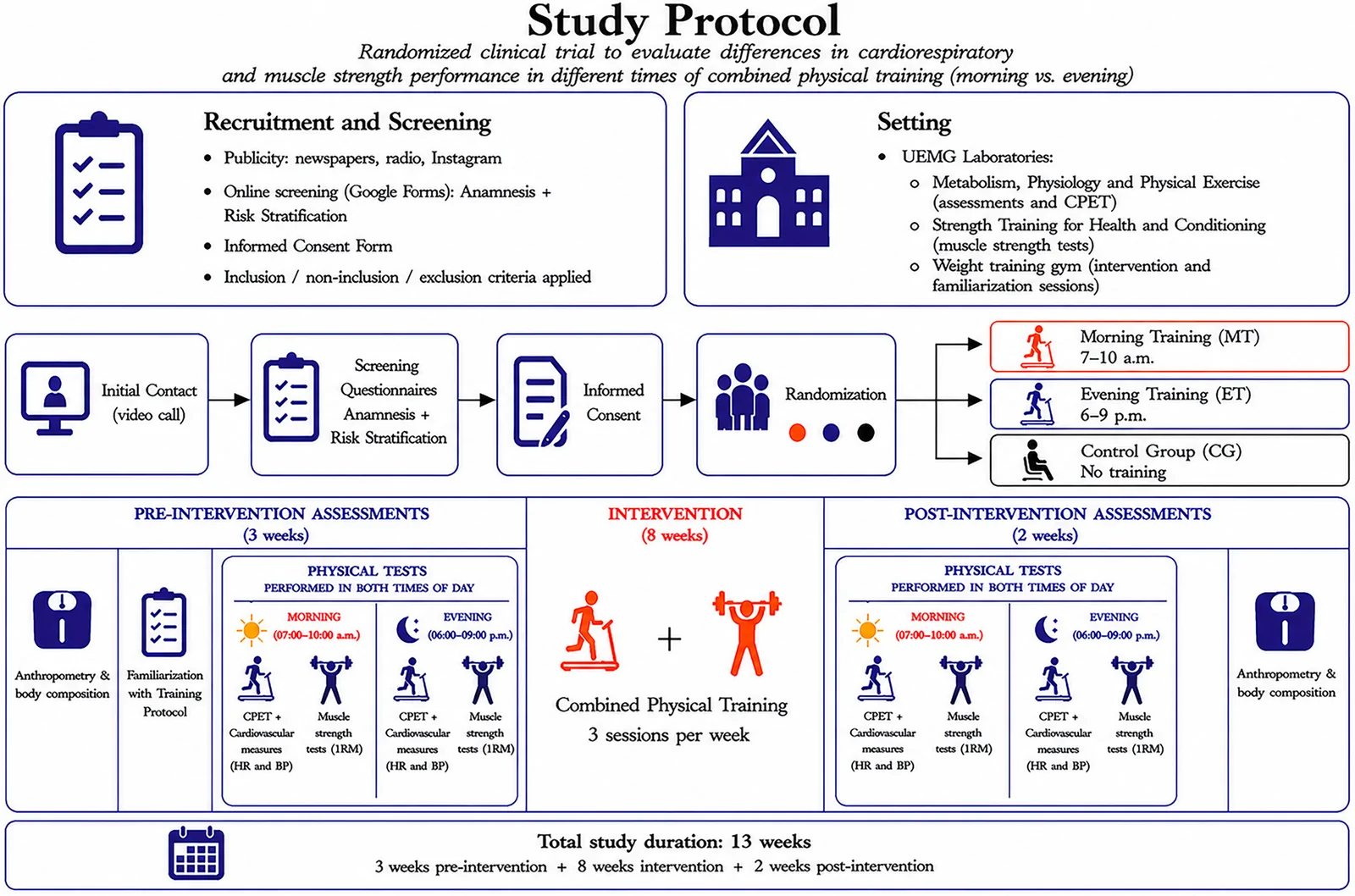
Study protocols.

In the present study, participants were instructed to maintain their habitual dietary intake, sleep routines, and lifestyle behaviors throughout the intervention period. Dietary intake was monitored using 24 h dietary recalls, whereas habitual activity patterns and physical activity levels were assessed by actigraphy before, during, and after the intervention (data not shown). However, these variables were collected primarily for monitoring purposes and were not pre-specified as primary or secondary outcomes of the present study. Given the pilot nature of the trial and the final sample size, no additional *post hoc* quantitative analyses of these variables were performed to avoid increasing analytical multiplicity and the potential for overinterpretation, concerns previously highlighted in randomized clinical trials (18). Furthermore, the inclusion of multiple lifestyle-related covariates in the mixed-effects models could increase the risk of model overfitting, particularly in studies with relatively small sample sizes (19).

### 2.4 Anamnesis

Using a Google Forms questionnaire, participants provided information regarding age, medical history, medication use, exercise habits, weight loss strategies, menstrual cycle characteristics, smoking status, alcohol consumption, and history of bariatric surgery.

### 2.5 Risk stratification form

The Exercise Preparticipation Health Screening Questionnaire for Exercise Professionals is a screening instrument designed to identify potential risks associated with participation in physical exercise and to determine whether an individual can safely engage in exercise or requires prior medical evaluation. This questionnaire was developed by the American College of Sports Medicine (16, 17). The screening process is organized into three stages. The first stage identifies individuals at elevated cardiovascular risk, for whom a positive response indicates the need for medical evaluation before initiating an exercise program. The second stage assesses current physical activity and exercise participation levels, whereas the third stage evaluates the presence of additional medical conditions. Individuals presenting such conditions, but who demonstrate an adequate level of physical activity, may be considered eligible to participate in light- to moderate-intensity exercise.

### 2.6 Circadian preference: morningness-eveningness questionnaire (HO)

The Morningness-Eveningness Questionnaire (20) was used to classify the circadian preference, in an adapted version for the Brazilian population (21). This consists of 19 questions regarding the individual's habitual daily life situations. The results classify individuals into five chronotype categories: evening (16–30 points); moderately evening (31–41 points); indifferent or intermediate (42–58 points); moderately morning (59–69 points); and morning (70–86 points).

### 2.7 Anthropometric measurements

Body mass was measured in kilograms (kg) using an InBody® 770 scale, and height was measured in centimeters using a stadiometer. Body mass index (BMI; kg/m^2^) was calculated as body mass (kg) divided by height squared (m^2^). Waist and hip circumferences were measured using a non-elastic measuring tape with a precision of 0.1 cm. Waist circumference was assessed with participants standing upright and the measuring tape positioned horizontally at the level of the umbilicus (17). A waist circumference ≥88 cm was used as the cutoff point for increased risk of obesity-related comorbidities, including type 2 diabetes, hypertension, and cardiovascular disease, in women (22). Hip circumference was measured with participants in the standing position, with the tape placed horizontally at the point of maximal gluteal protrusion (17).

### 2.8 Body composition

Body composition was assessed using tetrapolar multifrequency bioelectrical impedance analysis (InBody® 770). Participants were instructed to wear light clothing and remove all metallic objects and accessories before the assessment. Measurements were obtained with participants in an upright position, with the arms slightly abducted while holding the hand electrodes and the feet positioned on the footplate electrodes. The assessment was completed within approximately 1 min. To minimize potential sources of measurement variability, participants were instructed to avoid water consumption for at least 2 h before the assessment, undergo a minimum 4-h fasting period, and refrain from physical exercise, alcohol consumption, and caffeine intake for at least 24 h before testing, in accordance with the manufacturer's recommendations (InBody®).

### 2.9 Cardiopulmonary performance

Participants underwent cardiopulmonary exercise testing (CPET) to assess peak oxygen uptake (VO_2peak_) on a Super ATL treadmill (Inbramed®) while wearing a face mask connected to a Quark CPET metabolic cart (COSMED®, Rome, Italy). According to the ACSM preparticipation screening guidelines, this protocol is classified as not requiring physician supervision for individuals categorized as Stage 1 risk (17), indicating no additional cardiovascular risk among participants who did not present positive responses during the initial screening process. Importantly, this classification pertains exclusively to the level of medical supervision required and not to the intensity of the exercise test itself, as all participants performed the CPET until volitional exhaustion rather than to a predetermined submaximal workload or heart rate threshold.

The exercise protocol was adapted from La Scala Teixeira et al. (23). Participants began walking at a speed of 3 km/h, with increments of 1 km/h applied every 3 min until volitional fatigue. Perceived exertion was monitored throughout the test using the Borg Category-Ratio Scale (CR10), ranging from 0 to 10, with a score of 10 representing maximal perceived exertion (24). Participants were allowed to terminate the test at any time but were encouraged to continue until maximal volitional exhaustion. After test completion, a 3 min active recovery was performed at a walking speed of 3 km/h.

Accordingly, the primary outcome was reported as VO_2peak_ rather than maximal oxygen uptake (VO_2max_), consistent with the terminology recommended for symptom-limited incremental exercise tests that do not incorporate a VO_2_ plateau or secondary verification criteria (e.g., respiratory exchange ratio ≥1.10 or heart rate approaching the age-predicted maximum) required to confirm a true VO_2max_ (25). Following completion of the CPET, the investigator reviewed the VO_2peak_ response throughout the test and identified the 1-min interval with the highest recorded VO_2_ value for each participant, which was defined as VO_2peak_ (26).

### 2.10 Cardiovascular measurements

Participants performed the CPET while wearing an H10 heart rate monitor (Polar®, Oulu, Finland), positioned below the xiphoid process and paired with the Omnia software (COSMED®) for the simultaneous monitoring of metabolic and heart rate responses throughout the test. In addition, participants wore an M30 watch (Polar®, Oulu, Finland) on the wrist during the assessment. Systolic blood pressure (SBP) and diastolic blood pressure (DBP) were measured using a sphygmomanometer and stethoscope (Premium®, China) positioned over the brachial artery, in accordance with the recommendations of the Brazilian Guidelines for Blood Pressure Measurement Inside and Outside the Office—2023 (27).

### 2.11 Muscle strength performance

Participants performed a multiple-repetition test in the bench press and leg press exercises on the fifth familiarization session to estimate maximal strength using the Brzycki equation (28) before undertaking the one-repetition maximum (1RM) test. This procedure was adapted to improve the accuracy of maximal strength determination. During the multiple-repetition test, participants were instructed to perform the maximum number of repetitions possible using the loads selected during the familiarization sessions (sessions 2–4), which were established according to individual tolerance. Because the training protocol prescribed 16–20 repetitions during the first two weeks of the intervention, participants who exceeded 20 repetitions in a given exercise rested for 5 min before repeating the procedure with a higher load. This process was repeated until an appropriate load was identified at which the participant was unable to perform more than 20 repetitions.

Maximal strength was subsequently assessed using the 1RM test, defined as the maximum load that can be lifted once through the full range of motion of a given exercise. Following procedures described by Lacerda et al. (29, 30), participants performed 10 unloaded repetitions as a warm-up before testing. The initial load for the bench press and 90° leg press exercises was based on the estimated 1RM values obtained during the sixth familiarization session. During the 1RM test, the load was progressively increased after each successful attempt, and maximal strength was determined during the concentric phase of the movement. Participants were allowed a maximum of six attempts, with 3-min rest intervals between attempts (29, 30).

### 2.12 Combined training protocol

Participants assigned to the intervention groups completed an eight-week combined training program performed three times per week, totaling 24 sessions. They were allocated to either the MT group (7:00–10:00 a.m.) or the ET group (6:00–9:00 p.m.). Both groups performed the same combined training protocol, consisting of resistance and aerobic exercise. The duration of each exercise modality was matched within each training session, comprising 20 min of resistance exercise and 20 min of aerobic exercise during weeks 1–2 (total session duration: 40 min), 25 min of each modality during weeks 3–5 (50 min sessions), and 30 min of each modality during weeks 6–8 (60 min sessions). Thus, the intervention incorporated a progressive increase in both training volume and intensity throughout the study period. Participants were required to attend at least 85% of the prescribed sessions (i.e., a minimum of 20 sessions) to be included in the final analyses.

The resistance training protocol included the following exercises: free-weight bench press, 45^°^ leg press, seated row with a semi-pronated grip, leg curl, dumbbell lateral raise, and knee extension. Training volume ranged from two to four sets performed within a target repetition range of 8–20 repetitions maximum (RM). During weeks 1–2, participants performed two sets of 16–20 RM. During weeks 3–5, training volume was increased to three sets of 12–16 RM, whereas during weeks 6–8, participants completed four sets of 8–12 RM. Rest intervals were set at 60 s during weeks 1–2, 90 s during weeks 3–5, and 105 s during weeks 6–8. Each repetition lasted approximately four seconds, and sets were terminated when participants were unable to complete the concentric phase of the movement within the prescribed range of motion or repetition cadence. The resistance training program was designed according to the American College of Sports Medicine recommendations for muscle hypertrophy (31).

Aerobic training was performed on an 820 EXi motorized treadmill (EMBREEX®). Exercise intensity was prescribed based on peak oxygen uptake (VO_2peak_) values obtained during cardiopulmonary exercise testing (32). The aerobic protocol involved a progressive increase in both duration and intensity, ranging from 20 to 30 min per session and from 55% to 75% of VO_2peak_. During weeks 1–2, participants exercised at 55% of VO_2peak_. This intensity was increased to 65% during weeks 3–5 and to 75% during weeks 6–8.

### 2.13 Statistical analysis

Data were analyzed using R software (R Foundation for Statistical Computing, Vienna, Austria) within the RStudio integrated development environment. Continuous variables were summarized using descriptive statistics according to group (CG, MT, and ET), experimental period (pre- and post-intervention), testing time (morning or evening), and measurement moment (rest and post-exercise), as applicable. Inferential analyses were performed using linear mixed-effects models fitted by restricted maximum likelihood estimation. Participant was included as a random intercept in all models to account for the correlation between repeated measurements within individuals.

Anthropometric and body-composition outcomes were analyzed using models including group, experimental period, and their interaction as fixed effects. Physical-performance outcomes were analyzed using models including group, experimental period, testing time, and all two- and three-way interaction terms. Cardiovascular outcomes were analyzed using models including group, experimental period, testing time, measurement moment, and all corresponding two-, three-, and four-way interactions. Global fixed effects were evaluated using Type III *F*-tests with Satterthwaite-approximated degrees of freedom.

Estimated marginal means were calculated using the *emmeans* package, with Kenward-Roger degrees of freedom. Pre-to-post changes were estimated within each group and, when applicable, within each testing time and measurement moment. Differences in pre-to-post changes among groups were examined using interaction contrasts. Between-group pairwise comparisons were adjusted using Tukey's method, whereas interaction contrasts comparing pre-to-post changes among groups were adjusted using Sidak's method. No additional multiplicity adjustment was applied to single pre-to-post contrasts estimated within each group and testing condition. Results are presented as estimated mean differences with 95% confidence intervals and *p*-values.

Standardized effect sizes were calculated from model-estimated contrasts using the *eff_size*() function in the *emmeans* package. Cohen's *d* was obtained by dividing each estimated mean difference by the residual standard deviation of the corresponding fitted model. Confidence intervals for standardized effect sizes were calculated using model-based uncertainty and the residual degrees of freedom. Statistical significance was set at *p* < 0.05.

The analyses followed a per-protocol approach because only participants who completed the intervention and attended at least 85% of the prescribed training sessions were included. Linear mixed-effects models used all available observations from these participants; therefore, participants with an incomplete measurement at a specific assessment were not necessarily excluded from the entire analysis. The R packages, model specifications, analysis scripts, and corresponding references are provided in Supplementary Material 1.

## 3 Results

### 3.1 Exploratory sample characterization based on adiposity measures and circadian preference

The study was completed by 49 women with obesity, distributed as follows (Table 1): CG [*n* = 13; age: 35 ± 9 years; body fat percentage (BF%): 42.15 ± 6.45%], MT (*n* = 17; age: 39 ± 8 years; BF%: 44.42 ± 4.74%), and ET (*n* = 19; age: 39 ± 8 years; BF%: 44.90 ± 4.90%). Figure 3 shows the exploratory overview of sample circadian preferences: morning- and intermediate-types. None of the participants showed an evening preference.

**Table 1:** Characterization of a sample of women with obesity regarding anthropometry and body composition before and after 8 weeks of combined training at different times of the day.

	Control (CG, *n* = 13)		Morning Training (MT, *n* = 17)		Evening Training (ET, *n* = 19)		
Variable	Pre	Post	Pre	Post	Pre	Post	p
Body mass (kg)	87.9 ± 23.3	89.0 ± 23.1	94.0 ± 14.3	94.6 ± 14.4	89.2 ± 13.1	89.3 ± 13.4	0.240
Height (m)	1.64 ± 0.1	1.64 ± 0.1	1.65 ± 0.1	1.65 ± 0.1	1.64 ± 0.04	1.64 ± 0.04	-
BMI (kg/m^2^)	32.6 ± 6.5	33.0 ± 6.3	34.6 ± 4.2	34.8 ± 4.4	33.3 ± 4.3	33.3 ± 4.5	0.258
Waist circumference (cm)	99.2 ± 14.4*	102.2 ± 14.6	108.4 ± 13.9	107.5 ± 13.1	103.4 ± 12.5	102.9 ± 11.4	0.003*
Hip circumference (cm)	114.2 ± 15.7	117.9 ± 10.8	121.5 ± 9.6	122.4 ± 9.4	117.6 ± 7.9	118.8 ± 7.0	0.419
Body fat percentage (%)	42.1 ± 6.4	42.1 ± 6.1	44.4 ± 4.7	44.3 ± 5.0	44.9 ± 4.9	44.6 ± 4.2	0.897
Fat mass (kg)	38.0 ± 15.0	38.4 ± 14.2	42.0 ± 8.9	42.2 ± 9.4	40.4 ± 9.2	40.3 ± 9.4	0.696
Visceral fat area (cm^2^)	180.7 ± 62.0	181.1 ± 60.5	208.0 ± 39.5	205.8 ± 42.4	204.4 ± 34.4	203.1 ± 40.1	0.728
Skeletal muscle mass (kg)	27.7 ± 5.6	28.2 ± 5.8	28.2 ± 2.9	28.5 ± 2.7	26.9 ± 3.2	27.2 ± 3.2	0.510
Lean mass (kg)	47.0 ± 9.0	47.7 ± 9.5	47.7 ± 4.7	48.2 ± 4.4	46.0 ± 5.0	46.2 ± 5.0	0.480
Fat-free mass (kg)	49.9 ± 9.6	50.7 ± 10.1	50.7 ± 5.0	51.2 ± 4.7	48.8 ± 5.3	49.1 ± 5.3	0.498
Total body water (L)	36.5 ± 7.1	37.1 ± 7.4	37.1 ± 3.7	37.5 ± 3.4	35.8 ± 3.9	36.0 ± 3.9	0.462

Data presented as mean ± standard deviation. *p*-value represents the statistical analysis between the time factor experimental period: (pre- vs post-combined training) and the groups. The symbol “*” indicates an intragroup difference before and after the intervention. No difference was observed between groups at baseline. Only the control group exhibited within-group changes (pre- vs. post-intervention), which increased waist circumference (*p* < 0.05). Statistical analysis: Linear mixed-effects models with Tukey *post hoc* test (*p* < 0.05).

**Figure 3:**
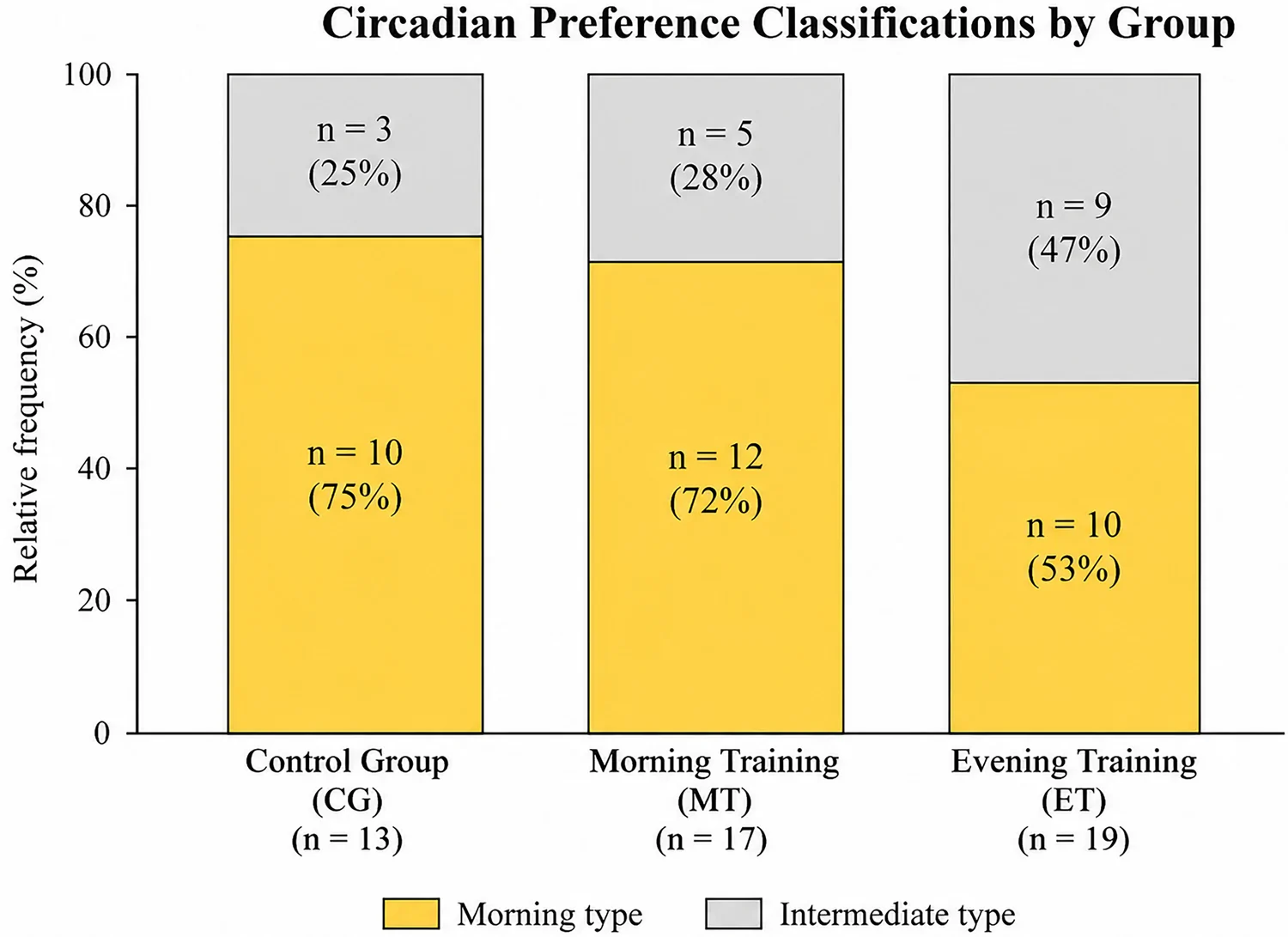
Circadian preference of the participants.

### 3.2 Body composition changes were limited and did not differ between training times

No between-group differences were observed at baseline for anthropometric or body composition variables (Table 2). As shown in Tables 1 and 3, significant main effects of the experimental period were observed only for waist circumference (*p* < 0.05). No significant group-by-period interactions were observed for these variables, indicating that the magnitude of the change did not differ among the CG, MT, and ET groups. Therefore, such changes could not be specifically attributed to training performed in the morning or in the evening. Waist circumference showed a significant group-by-period interaction, primarily reflecting an increase of 3.01 cm in CG (95% CI: 1.20–4.82; *p* = 0.002; *d* = 1.31). No significant changes were observed in MT [−0.99 cm (95% CI: −2.57 to 0.59; *p* = 0.212; *d* = −0.43)] or ET [−0.47 cm (95% CI: −1.97 to 1.02; *p* = 0.527; *d* = −0.21)]. Body fat percentage, fat mass, and visceral fat area showed no significant main effects of experimental period or group-by-period interactions.

**Table 2:** Baseline comparisons between the three experimental groups of women with obesity regarding anthropometry and body composition.

Variable	M ± SD	Contrast (pre-intervention)	*p*-value
Body mass (kg)	87.9 ± 23.3 vs. 94.0 ± 14.3	Control pre-intervention - Morning Training pre-intervention	0.592
Body mass (kg)	87.9 ± 23.3 vs. 89.2 ± 13.1	Control pre-intervention - Evening Training pre-intervention	0.974
Body mass (kg)	94.0 ± 14.3 vs. 89.2 ± 13.1	Morning Training pre-intervention - Evening Training pre-intervention	0.675
Height (m)	1.64 ± 0.1 vs. 1.65 ± 0.1	Control pre-intervention - Morning Training pre-intervention	-
Height (m)	1.64 ± 0.1 vs. 1.64 ± 0.04	Control pre-intervention - Evening Training pre-intervention	-
Height (m)	1.65 ± 0.1 vs. 1.64 ± 0.04	Morning Training pre-intervention - Evening Training pre-intervention	-
BMI (kg/m^2^)	32.6 ± 6.5 vs. 34.6 ± 4.2	Control pre-intervention - Morning Training pre-intervention	0.538
BMI (kg/m^2^)	32.6 ± 6.5 vs. 33.3 ± 4.3	Control pre-intervention - Evening Training pre-intervention	0.926
BMI (kg/m^2^)	34.6 ± 4.2 vs. 33.3 ± 4.3	Morning Training pre-intervention - Evening Training pre-intervention	0.720
Waist circumference (cm)	99.2 ± 14.4 vs. 108.4 ± 13.9	Control pre-intervention - Morning Training pre-intervention	0.148
Waist circumference (cm)	99.2 ± 14.4 vs. 103.4 ± 12.5	Control pre-intervention - Evening Training pre-intervention	0.649
Waist circumference (cm)	108.4 ± 13.9 vs. 103.4 ± 12.5	Morning Training pre-intervention - Evening Training pre-intervention	0.491
Hip circumference (cm)	114.2 ± 15.7 vs. 121.5 ± 9.6	Control pre-intervention - Morning Training pre-intervention	0.126
Hip circumference (cm)	114.2 ± 15.7 vs. 117.6 ± 7.9	Control pre-intervention - Evening Training pre-intervention	0.603
Hip circumference (cm)	121.5 ± 9.6 vs. 117.6 ± 7.9	Morning Training pre-intervention - Evening Training pre-intervention	0.488
Body fat percentage (%)	42.1 ± 6.4 vs. 44.4 ± 4.7	Control pre-intervention - Morning Training pre-intervention	0.444
Body fat percentage (%)	42.1 ± 6.4 vs. 44.9 ± 4.9	Control pre-intervention - Evening Training pre-intervention	0.296
Body fat percentage (%)	44.4 ± 4.7 vs. 44.9 ± 4.9	Morning Training pre-intervention - Evening Training pre-intervention	0.960
Fat mass (kg)	38.0 ± 15.0 vs. 42.0 ± 8.9	Control pre-intervention - Morning Training pre-intervention	0.591
Fat mass (kg)	38.0 ± 15.0 vs. 40.4 ± 9.2	Control pre-intervention - Evening Training pre-intervention	0.820
Fat mass (kg)	42.0 ± 8.9 vs. 40.4 ± 9.2	Morning Training pre-intervention - Evening Training pre-intervention	0.900
Visceral fat area (cm^2^)	180.7 ± 62.0 vs. 208.0 ± 39.5	Control pre-intervention - Morning Training pre-intervention	0.250
Visceral fat area (cm^2^)	180.7 ± 62.0 vs. 204.4 ± 34.4	Control pre-intervention - Evening Training pre-intervention	0.332
Visceral fat area (cm^2^)	208.0 ± 39.5 vs. 204.4 ± 34.4	Morning Training pre-intervention - Evening Training pre-intervention	0.970
Skeletal muscle mass (kg)	27.7 ± 5.6 vs. 28.2 ± 2.9	Control pre-intervention - Morning Training pre-intervention	0.948
Skeletal muscle mass (kg)	27.7 ± 5.6 vs. 26.9 ± 3.2	Control pre-intervention - Evening Training pre-intervention	0.848
Skeletal muscle mass (kg)	28.2 ± 2.9 vs. 26.9 ± 3.2	Morning Training pre-intervention - Evening Training pre-intervention	0.621
Lean mass (kg)	47.0 ± 9.0 vs. 47.7 ± 4.7	Control pre-intervention - Morning Training pre-intervention	0.939
Lean mass (kg)	47.0 ± 9.0 vs. 46.0 ± 5.0	Control pre-intervention - Evening Training pre-intervention	0.898
Lean mass (kg)	47.7 ± 4.7 vs. 46.0 ± 5.0	Morning Training pre-intervention - Evening Training pre-intervention	0.674
Fat-free mass (kg)	49.9 ± 9.6 vs. 50.7 ± 5.0	Control pre-intervention - Morning Training pre-intervention	0.946
Fat-free mass (kg)	49.9 ± 9.6 vs. 48.8 ± 5.3	Control pre-intervention - Evening Training pre-intervention	0.900
Fat-free mass (kg)	50.7 ± 5.0 vs. 48.8 ± 5.3	Morning Training pre-intervention - Evening Training pre-intervention	0.693
Total body water (L)	36.5 ± 7.1 vs. 37.1 ± 3.7	Control pre-intervention - Morning Training pre-intervention	0.937
Total body water (L)	36.5 ± 7.1 vs. 35.8 ± 3.9	Control pre-intervention - Evening Training pre-intervention	0.913
Total body water (L)	37.1 ± 3.7 vs. 35.8 ± 3.9	Morning Training pre-intervention - Evening Training pre-intervention	0.694

Baseline comparisons between the three experimental groups (Control, Morning Training, Evening Training) of women with obesity regarding anthropometry and body composition. Values are presented as mean ± SD. *p*-value represents the comparison between the groups (Tukey-adjusted). Statistical analysis: linear mixed-effects models with Tukey's *post hoc* test (*p* < 0.05).

**Table 3:** Model-based effect sizes and 95% confidence intervals for pre-to-post changes in anthropometric and body-composition outcomes.

Variable	Control (CG, *n* = 13)	Morning training (MT, *n* = 17)	Evening training (ET, *n* = 19)
Body mass (kg)	0.91 (0.11 to 1.71)	0.52 (−0.17 to 1.22)	0.05 (−0.60 to 0.70)
BMI (kg/m^2^)	0.88 (0.07 to 1.68)	0.57 (−0.12 to 1.27)	0.05 (−0.60 to 0.71)
Waist circumference (cm)	1.31 (0.50 to 2.13)	−0.43 (−1.13 to 0.26)	−0.21 (−0.86 to 0.45)
Hip circumference (cm)	0.84 (0.04 to 1.63)	0.20 (−0.49 to 0.89)	0.26 (−0.39 to 0.91)
Body fat percentage (%)	−0.04 (−0.83 to 0.75)	−0.17 (−0.86 to 0.52)	−0.28 (−0.93 to 0.38)
Fat mass (kg)	0.27 (−0.52 to 1.06)	0.17 (−0.52 to 0.86)	−0.14 (−0.79 to 0.52)
Visceral fat area (cm^2^)	0.06 (−0.73 to 0.85)	−0.35 (−1.04 to 0.34)	−0.21 (−0.86 to 0.45)
Skeletal muscle mass (kg)	1.08 (0.28 to 1.89)	0.69 (−0.01 to 1.39)	0.49 (−0.17 to 1.15)
Lean mass (kg)	0.95 (0.15 to 1.76)	0.62 (−0.08 to 1.31)	0.33 (−0.32 to 0.99)
Fat-free mass (kg)	0.92 (0.12 to 1.72)	0.59 (−0.10 to 1.29)	0.31 (−0.34 to 0.97)
Total body water (L)	0.92 (0.12 to 1.72)	0.58 (−0.11 to 1.28)	0.28 (−0.37 to 0.94)

Values are presented as Cohen's *d* effect sizes with 95% confidence intervals for model-based within-group post-vs.-pre comparisons. Effect sizes were calculated from estimated marginal means and standardized using the residual standard deviation of the corresponding linear mixed-effects model. Positive values indicate increases, and negative values indicate decreases from pre- to post-intervention.

### 3.3 Upper- and lower-limb strength improved in both training groups

No between-group differences were observed at baseline for physical fitness variables (Table 4). Significant group-by-period interactions were observed for both bench-press and leg-press 1RM (Figure 4, Tables 5, 6). However, no significant group-by-period-by-testing-time interactions were identified, indicating that differences in adaptation among groups did not consistently depend on whether testing was performed in the morning or evening. Bench-press 1RM increased in MT by 5.79 kg during morning testing (95% CI: 4.37–7.21; *p* < 0.001; *d* = 2.76) and by 5.31 kg during evening testing (95% CI: 3.86–6.76; *p* < 0.001; *d* = 2.53). Corresponding increases in ET were 4.97 kg during morning testing (95% CI: 3.57–6.36; *p* < 0.001; *d* = 2.37) and 5.50 kg during evening testing (95% CI: 4.10–6.89; *p* < 0.001; *d* = 2.62). No significant changes were observed in CG. When testing times were averaged, the pre-to-post increase in bench-press 1RM was 5.14 kg greater in MT than in CG (95% CI: 3.06–7.21; *p* < 0.001) and 4.82 kg greater in ET than in CG (95% CI: 2.77–6.87; *p* < 0.001), with no significant difference between MT and ET.

**Table 4:** Baseline comparisons between the three experimental groups of women with obesity regarding cardiopulmonary performance and maximal strength.

Variable	M ± SD	Time of testing	Contrast (pre-intervention)	*p*-value
VO_2peak_ (mL·kg^−1^·min^−1^)	24 ± 5 vs. 21 ± 3	Morning	Control pre-intervention—Morning Training pre-intervention	0.178
VO_2peak_ (mL·kg^−1^·min^−1^)	24 ± 5 vs. 23 ± 4	Morning	Control pre-intervention—Evening Training pre-intervention	0.897
VO_2peak_ (mL·kg^−1^·min^−1^)	21 ± 3 vs. 23 ± 4	Morning	Morning Training pre-intervention—Evening Training pre-intervention	0.280
VO_2peak_ (mL·kg^−1^·min^−1^)	25 ± 5 vs. 21 ± 3	Evening	Control pre-intervention—Morning Training pre-intervention	0.059
VO_2peak_ (mL·kg^−1^·min^−1^)	25 ± 5 vs. 23 ± 4	Evening	Control pre-intervention—Evening Training pre-intervention	0.372
VO_2peak_ (mL·kg^−1^·min^−1^)	21 ± 3 vs. 23 ± 4	Evening	Morning Training pre-intervention—Evening Training pre-intervention	0.497
Duration (min)	16 ± 4 vs. 14 ± 3	Morning	Control pre-intervention—Morning Training pre-intervention	0.194
Duration (min)	16 ± 4 vs. 14 ± 2	Morning	Control pre-intervention—Evening Training pre-intervention	0.305
Duration (min)	14 ± 3 vs. 14 ± 2	Morning	Morning Training pre-intervention—Evening Training pre-intervention	0.940
Duration (min)	16 ± 4 vs. 14 ± 3	Evening	Control pre-intervention—Morning Training pre-intervention	0.259
Duration (min)	16 ± 4 vs. 14 ± 2	Evening	Control pre-intervention—Evening Training pre-intervention	0.324
Duration (min)	14 ± 3 vs. 14 ± 2	Evening	Morning Training pre-intervention—Evening Training pre-intervention	0.980
Maximum velocity (km/h)	8 ± 1 vs. 7 ± 1	Morning	Control pre-intervention—Morning Training pre-intervention	0.586
Maximum velocity (km/h)	8 ± 1 vs. 7 ± 1	Morning	Control pre-intervention—Evening Training pre-intervention	0.783
Maximum velocity (km/h)	7 ± 1 vs. 7 ± 1	Morning	Morning Training pre-intervention—Evening Training pre-intervention	0.923
Maximum velocity (km/h)	7 ± 1 vs. 7 ± 1	Evening	Control pre-intervention—Morning Training pre-intervention	0.513
Maximum velocity (km/h)	7 ± 1 vs. 7 ± 1	Evening	Control pre-intervention—Evening Training pre-intervention	0.842
Maximum velocity (km/h)	7 ± 1 vs. 7 ± 1	Evening	Morning Training pre-intervention—Evening Training pre-intervention	0.803
Upper limbs maximum strength (kg)	22 ± 7 vs. 23 ± 4	Morning	Control pre-intervention—Morning Training pre-intervention	0.979
Upper limbs maximum strength (kg)	22 ± 7 vs. 18 ± 6	Morning	Control pre-intervention—Evening Training pre-intervention	0.171
Upper limbs maximum strength (kg)	23 ± 4 vs. 18 ± 6	Morning	Morning Training pre-intervention—Evening Training pre-intervention	0.063
Upper limbs maximum strength (kg)	21 ± 6 vs. 22 ± 4	Evening	Control pre-intervention—Morning Training pre-intervention	0.883
Upper limbs maximum strength (kg)	21 ± 6 vs. 18 ± 6	Evening	Control pre-intervention—Evening Training pre-intervention	0.248
Upper limbs maximum strength (kg)	22 ± 4 vs. 18 ± 6	Evening	Morning Training pre-intervention—Evening Training pre-intervention	0.053
Lower limbs maximum strength (kg)	181 ± 53 vs. 188 ± 44	Morning	Control pre-intervention—Morning Training pre-intervention	0.903
Lower limbs maximum strength (kg)	181 ± 53 vs. 159 ± 46	Morning	Control pre-intervention—Evening Training pre-intervention	0.402
Lower limbs maximum strength (kg)	188 ± 44 vs. 159 ± 46	Morning	Morning Training pre-intervention—Evening Training pre-intervention	0.129
Lower limbs maximum strength (kg)	167 ± 41 vs. 184 ± 47	Evening	Control pre-intervention—Morning Training pre-intervention	0.604
Lower limbs maximum strength (kg)	167 ± 41 vs. 160 ± 37	Evening	Control pre-intervention—Evening Training pre-intervention	0.784
Lower limbs maximum strength (kg)	184 ± 47 vs. 160 ± 37	Evening	Morning Training pre-intervention—Evening Training pre-intervention	0.158

Baseline comparisons between the three experimental groups (Control, Morning Training, Evening Training) of women with obesity regarding cardiopulmonary performance and maximal strength, stratified by morning and evening testing sessions. Values are presented as mean ± SD. *p*-value represents the comparison between the groups (Tukey-adjusted). Statistical analysis: linear mixed-effects models with Tukey's *post hoc* test (*p* < 0.05).

**Figure 4:**
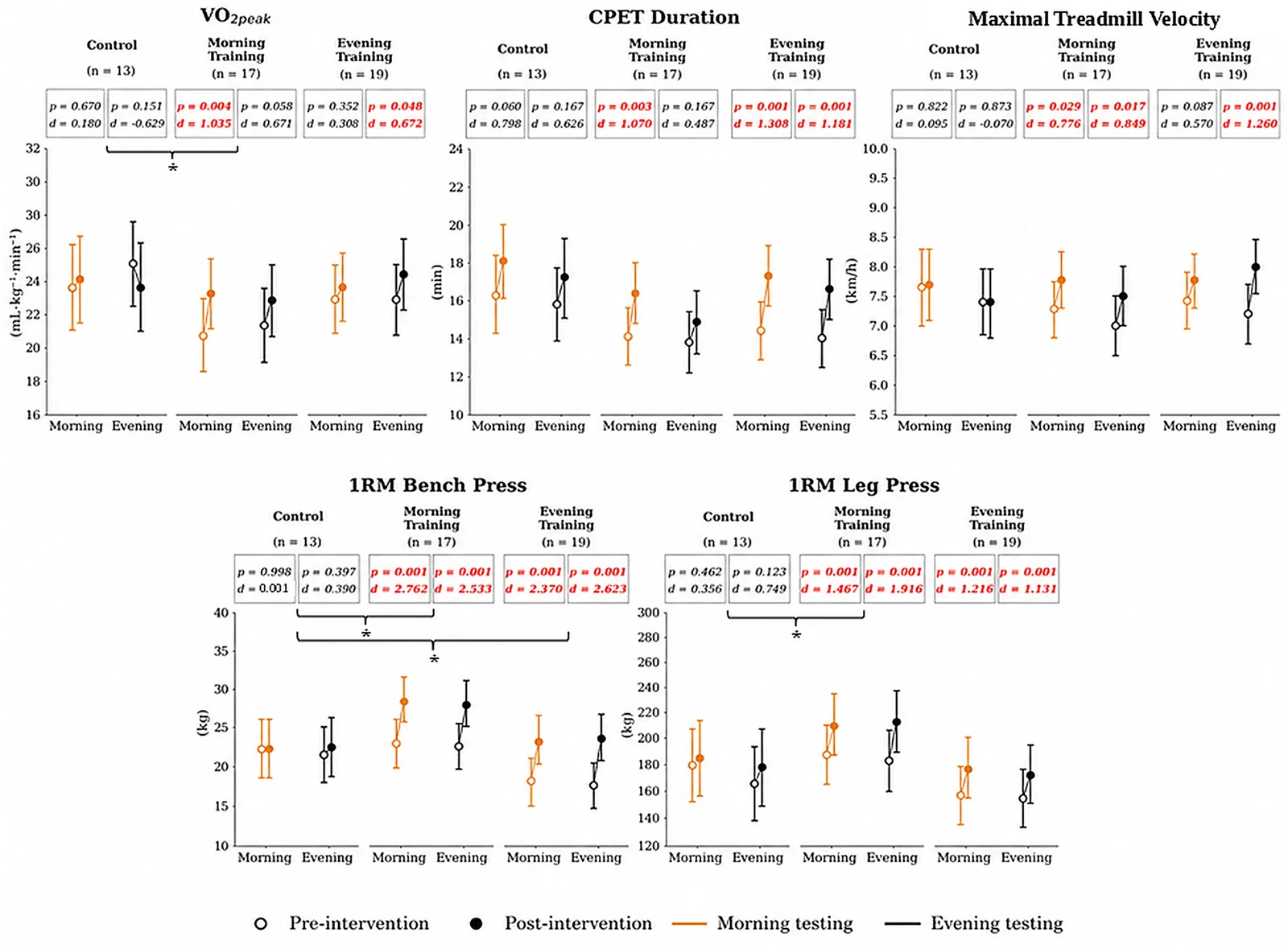
Estimated marginal means and 95% confidence intervals for VO₂_peak_, CPET duration, maximal treadmill velocity, bench-press 1RM, and leg-press 1RM before and after the eight-week intervention in women with obesity. Estimates were derived from linear mixed-effects models including group, experimental period, testing time, and their interactions, with participants included as a random effect. The *p* values and Cohen's *d* effect sizes shown above each condition refer to model-based within-group contrasts comparing post- versus pre-intervention values separately for morning and evening testing. Adjustments for multiple comparisons were performed using Tukey's or Sidak's method, as appropriate. Significant contrasts (*p* < 0.05) are highlighted in red; “*” symbols represent group-by-period interaction (pre- vs. post-intervention). CG, control group; MT, morning-training group; ET, evening-training group. Total sample: *n* = 49 (CG, *n* = 13; MT, *n* = 17; ET, *n* = 19).

**Table 5:** Cardiopulmonary performance and muscle strength of women with obesity before and after 8 weeks of combined training at different times of the day.

Variable		Control (CG, *n* = 13)					Morning training (MT, *n* = 17)						Evening training (ET, *n* = 19)						
	a.m.	a.m.		p.m.	p.m.		a.m.	a.m.		p.m.	p.m.		a.m.	a.m.		p.m.	p.m.		
	Pre	Post	Δ%	Pre	Post	Δ%	Pre	Post	Δ%	Pre	Post	Δ%	Pre	Post	Δ%	Pre	Post	Δ%	p
VO_2peak_ (mL·kg^−1^·min^−1^)	24 ± 5	24 ± 6	0%	25 ± 5	24 ± 4	−4%	21 ± 3*	23 ± 6*	10%	21 ± 3	23 ± 4	10%	23 ± 4	24 ± 5	4%	23 ± 4	25 ± 4	9%	0.281
Duration (min)	16 ± 4	18 ± 4	13%	16 ± 4	17 ± 4	6%	14 ± 3*	16 ± 3*	14%	14 ± 3	15 ± 4	7%	14 ± 2*	17 ± 4*	21%	14 ± 3	17 ± 3	21%	0.775
Maximum velocity (km/h)	8 ± 1	8 ± 1	0%	7 ± 1	7 ± 1	0%	7 ± 1*	8 ± 1*	14%	7 ± 1	8 ± 1	14%	7 ± 1*	8 ± 1*	14%	7 ± 1	8 ± 1	14%	0.476
Upper limbs maximum strength (kg)	22 ± 7	22 ± 7^a^	0%	21 ± 6	22 ± 7	5%	23 ± 4*	28 ± 5*	22%	22 ± 4	28 ± 5	27%	18 ± 6*	23 ± 6^*^^,^^a^	28%	18 ± 6	23 ± 6	28%	0.685
Lower limbs maximum strength (kg)	181 ± 53	171 ± 38	−6%	167 ± 41	164 ± 40	−2%	188 ± 44	207 ± 52*	10%	184 ± 47	208 ± 51	13%	159 ± 46*	179 ± 42^*^^,^^a^	13%	160 ± 37	172 ± 41	8%	0.711

Data presented as mean ± standard deviation. *p*-value: represents the interaction between the experimental period factor (pre- vs. post-combined training), the groups, and the time of day the test was performed.

a The symbol indicates between-group post-intervention difference compared to MT. Statistical analysis: Linear mixed effects models, with Tukey's *post hoc* test and Cohen's *d* effect size (*p* < 0.05). VO_2peak_: peak oxygen consumption.

* The symbol indicates an intragroup difference before and after the intervention.

**Table 6:** Cardiopulmonary performance and muscle strength with effect sizes and confidence intervals of pre- and post-intervention comparisons.

Variable	Control (CG, *n* = 13)		Morning training (MT, *n* = 17)		Evening training (ET, *n* = 19)	
	a.m.	p.m.	a.m.	p.m.	a.m.	p.m.
VO_2peak_ (mL·kg^−1^·min^−1^)	*d* = 0.18 (95% CI: −0.66, 1.02)	*d* = −0.63 (95% CI: −1.50, 0.24)	*d* = 1.04* (95% CI: 0.33, 1.74)	*d* = 0.67 (95% CI: −0.03, 1.37)	*d* = 0.31 (95% CI: −0.35, 0.97)	*d* = 0.67* (95% CI: 0.00, 1.35)
Duration (min)	*d* = 0.80 (95% CI: −0.04, 1.64)	*d* = 0.63 (95% CI: −0.27, 1.52)	*d* = 1.07* (95% CI: 0.36, 1.78)	*d* = 0.49 (95% CI: −0.21, 1.18)	*d* = 1.31* (95% CI: 0.64, 1.98)	*d* = 1.18* (95% CI: 0.51, 1.85)
Maximum velocity (km/h)	*d* = 0.09 (95% CI: −0.74, 0.93)	*d* = −0.07 (95% CI: −0.93, 0.80)	*d* = 0.78* (95% CI: 0.07, 1.48)	*d* = 0.85* (95% CI: 0.15, 1.55)	*d* = 0.57 (95% CI: −0.09, 1.23)	*d* = 1.26* (95% CI: 0.59, 1.93)
Upper limbs maximum strength (kg)	*d* = 0.00 (95% CI: −0.91, 0.92)	*d* = 0.39 (95% CI: −0.53, 1.31)	*d* = 2.76* (95% CI: 2.01, 3.51)	*d* = 2.53* (95% CI: 1.78, 3.29)	*d* = 2.37* (95% CI: 1.65, 3.09)	*d* = 2.62* (95% CI: 1.89, 3.36)
Lower limbs maximum strength (kg)	*d* = 0.36 (95% CI: −0.61, 1.32)	*d* = 0.75 (95% CI: −0.22, 1.72)	*d* = 1.47* (95% CI: 0.74, 2.19)	*d* = 1.92* (95% CI: 1.17, 2.67)	*d* = 1.22* (95% CI: 0.51, 1.92)	*d* = 1.13* (95% CI: 0.44, 1.82)

Values are presented as Cohen's *d* effect sizes with 95% confidence intervals for model-based within-group post-vs.-pre comparisons. Effect sizes were calculated from estimated marginal means and standardized using the residual deviation of the corresponding linear mixed-effects model. Positive values indicate increases, and negative indicate decreases from pre- to post-intervention.

The symbol “*” indicates an intragroup difference before and after the intervention.

Similarly, leg-press 1RM increased in MT by 22.20 kg during morning testing (95% CI: 11.69–32.70; *p* < 0.001; *d* = 1.47) and by 29.01 kg during evening testing (95% CI: 18.26–39.80; *p* < 0.001; *d* = 1.92). Leg-press 1RM also increased in ET by 18.40 kg during morning testing (95% CI: 8.12–28.70; *p* < 0.001; *d* = 1.22) and by 17.11 kg during evening testing (95% CI: 7.03–27.20; *p* = 0.001; *d* = 1.13). No significant changes were observed in CG. When testing times were averaged, the pre-to-post increase in leg-press 1RM was 17.24 kg greater in MT than in CG (95% CI: 1.36–33.13; *p* = 0.029), whereas ET did not differ significantly from either CG or MT.

### 3.4 Cardiopulmonary performance improved without consistent time-of-day superiority

Maximal treadmill velocity showed a significant main effect of experimental period (*p* < 0.001), but neither the group-by-period interaction (*p* = 0.051) nor the group-by-period-by-testing-time interaction (*p* = 0.476) was significant (Figure 4, Tables 5, 6). Within-group increases were observed in MT during morning testing [0.47 km·h^−1^ (95% CI: 0.05–0.90; *p* = 0.029; *d* = 0.78)] and evening testing [0.52 km·h^−1^ (95% CI: 0.09–0.94; *p* = 0.017; *d* = 0.85)], and in ET during evening testing [0.77 km·h^−1^ (95% CI: 0.37–1.17; *p* < 0.001; *d* = 1.26)]. The increase in ET during morning testing did not reach statistical significance [0.35 km·h^−1^ (95% CI: −0.05 to 0.75; *p* = 0.087; *d* = 0.57)], and no significant changes were observed in CG. Because direct comparisons of pre-to-post changes did not differ significantly among groups, these within-group findings do not indicate a differential effect of either training schedule.

A significant group-by-period interaction was observed for VO_2peak_, whereas the group-by-period-by-testing-time interaction was not significant. VO_2peak_ increased in MT during morning testing [2.42 mL·kg^−1^·min^−1^ (95% CI: 0.80–4.04; *p* = 0.004; *d* = 1.04)], whereas the change during evening testing did not reach significance [1.57 mL·kg^−1^·min^−1^ (95% CI: −0.05 to 3.19; *p* = 0.058; *d* = 0.67)]. In ET, VO_2peak_ increased during evening testing [1.57 mL·kg^−1^·min^−1^ (95% CI: 0.01–3.13; *p* = 0.048; *d* = 0.67)], but not during morning testing [0.72 mL·kg^−1^·min^−1^ (*p* = 0.352; *d* = 0.31)]. No significant changes were observed in CG. When testing times were averaged, the pre-to-post increase in VO_2peak_ was 2.52 mL·kg^−1^·min^−1^ greater in MT than in CG (95% CI: 0.29–4.75; *p* = 0.021), whereas ET did not differ significantly from CG or MT.

CPET duration also showed a significant main effect of the experimental period, indicating an overall increase from pre- to post-intervention. However, neither the group-by-period nor the group-by-period-by-testing-time interaction was significant (*p* > 0.05), indicating that the magnitude of change did not differ among groups or consistently depend on testing time. Although significant within-group increases were observed in MT during morning testing and in ET during both testing sessions, these isolated contrasts should not be interpreted as group-specific intervention effects in the absence of a significant group-by-period interaction.

### 3.5 Pre-to-post intervention changes in cardiovascular responses did not differ consistently among groups, testing times, or measurement moments

Significant main effects of measurement moment were observed for HR, SBP, and DBP (all *p* < 0.001), indicating significant differences between resting and immediately post-CPET values (Tables 7, 8). However, no significant higher-order interactions involving group, experimental period, testing time, and measurement moment were observed for HR, SBP, or DBP. These findings indicate that pre-to-post changes in resting and post-exercise cardiovascular responses did not differ consistently among groups or according to testing time.

**Table 7:** Cardiovascular responses before and after 8 weeks of combined training at different times of the day in women with obesity.

Variable	Control (CG, *n* = 13)						Morning training (MT, *n* = 17)						Evening training (ET, *n* = 19)						
	a.m.	a.m.		p.m.	p.m.		a.m.	a.m.		p.m.	p.m.		a.m.	a.m.		p.m.	p.m.		
	Pre	Post	Δ%	Pre	Post	Δ%	Pre	Post	Δ%	Pre	Post	Δ%	Pre	Post	Δ%	Pre	Post	Δ%	p
Resting HR (bpm)	82 ± 14	76 ± 12	−7%	80 ± 8	85 ± 11	6%	82 ± 13	79 ± 10	−4%	78 ± 10	81 ± 9	4%	82 ± 9	79 ± 13	−4%	81 ± 10	79 ± 10	−2%	0.082
Post-test HR (bpm)	160 ± 22^#^	161 ± 24^#^	1%	163 ± 20^#^	164 ± 25^#^	1%	147 ± 31^#^	164 ± 17^#^	12%	164 ± 23^#^	163 ± 2^#^	−1%	167 ± 13^#^	160 ± 24^#^	−4%	153 ± 28^#^	159 ± 30^#^	4%	
Resting SBP (mmHg)	113 ± 8	114 ± 12	1%	120 ± 11	116 ± 11	−3%	115 ± 13	112 ± 7	−3%	119 ± 14	116 ± 13	−3%	117 ± 11	114 ± 10	−3%	112 ± 8	112 ± 11	0%	0.077
Post-test SBP (mmHg)	179 ± 37^#^	148 ± 24^#^	−17%	180 ± 37^#^	176 ± 31^#^	−2%	175 ± 37^#^	174 ± 34^#^	−1%	190 ± 26^#^	173 ± 3^#^	−9%	179 ± 35^#^	173 ± 29^#^	−3%	171 ± 34^#^	190 ± 27^#^	11%	
Resting DBP (mmHg)	76 ± 9	75 ± 12	−1%	80 ± 12	79 ± 9	−1%	79 ± 9	76 ± 11	−4%	84 ± 11	81 ± 11	−4%	75 ± 10	80 ± 8	7%	81 ± 6	80 ± 6	−1%	0.374
Post-test DBP (mmHg)	83 ± 11^#^	78 ± 11^#^	−6%	93 ± 17^#^	87 ± 9^#^	−6%	85 ± 12^#^	90 ± 14^#^	6%	98 ± 14^#^	95 ± 13^#^	−3%	88 ± 16^#^	89 ± 10^#^	1%	91 ± 13^#^	90 ± 14^#^	−1%	

Data presented as mean ± standard deviation. *p*-value: represents the interaction between the factors of experimental period (pre- vs. post-combined training), groups, the time of day the test was performed, and measurement moment (rest and post-test).

# The symbol indicates intragroup difference at resting and post-CPET measures. Statistical analysis: linear mixed-effects models with Tukey's *post hoc* test (*p* < 0.05).

HR, heart rate; SBP, systolic blood pressure; DBP, diastolic blood pressure.

**Table 8:** Cardiovascular responses to combined training at different times of day in women with obesity, with effect sizes and confidence intervals of pre- and post-intervention comparisons.

Variable	Control (CG, *n* = 13)		Morning training (MT, *n* = 17)		Evening training (ET, *n* = 19)	
	a.m.	p.m.	a.m.	p.m.	a.m.	p.m.
Resting HR (bpm)	*d* = −0.35 (95% CI: −1.18, 0.47)	*d* = 0.33 (95% CI: −0.55, 1.20)	*d* = −0.21 (95% CI: −0.89, 0.48)	*d* = 0.17 (95% CI: −0.53, 0.87)	*d* = −0.17 (95% CI: −0.83, 0.49)	*d* = −0.18 (95% CI: −0.84, 0.48)
Post-test HR (bpm)	*d* = 0.11 (95% CI: −0.74, 0.95)	*d* = 0.12 (95% CI: −0.76, 0.99)	*d* = 0.94 (95% CI: 0.25, 1.63)	*d* = −0.04 (95% CI: −0.75, 0.68)	*d* = −0.40 (95% CI: −1.06, 0.25)	*d* = 0.38 (95% CI: −0.29, 1.04)
Resting SBP (mmHg)	*d* = 0.16 (95% CI: −0.67, 0.98)	*d* = −0.16 (95% CI: −1.04, 0.72)	*d* = −0.20 (95% CI: −0.88, 0.49)	*d* = −0.19 (95% CI: −0.88, 0.49)	*d* = −0.15 (95% CI: −0.81, 0.51)	*d* = 0.01 (95% CI: −0.64, 0.66)
Post-test SBP (mmHg)	*d* = −1.35 (95% CI: −2.18, −0.52)	*d* = −0.14 (95% CI: −1.02, 0.73)	*d* = −0.12 (95% CI: −0.81, 0.57)	*d* = −0.85 (95% CI: −1.54, −0.16)	*d* = −0.27 (95% CI: −0.92, 0.38)	*d* = 0.93 (95% CI: 0.25, 1.60)
Resting DBP (mmHg)	*d* = −0.12 (95% CI: −0.94, 0.71)	*d* = −0.21 (95% CI: −1.09, 0.67)	*d* = −0.33 (95% CI: −1.02, 0.36)	*d* = −0.24 (95% CI: −0.93, 0.44)	*d* = 0.48 (95% CI: −0.18, 1.14)	*d* = 0.01 (95% CI: −0.65, 0.67)
Post-test DBP (mmHg)	*d* = −0.55 (95% CI: −1.40, 0.30)	*d* = 0.53 (95% CI: −0.16, 1.22)	*d* = 0.04 (95% CI: −0.61, 0.69)	*d* = −0.69 (95% CI: −1.57, 0.19)	*d* = −0.28 (95% CI: −0.97, 0.41)	*d* = 0.01 (95% CI: −0.65, 0.67)

Values are presented as Cohen's *d* effect sizes with 95% confidence intervals for model-based within-group post-vs.-pre comparisons. Effect sizes were calculated from estimated marginal means and standardized using the residual standard deviation of the corresponding linear mixed-effects model. Positive values indicate increases, and negative values indicate decreases from pre- to post-intervention.

Although a small number of isolated pairwise contrasts reached statistical significance, these findings were not supported by the corresponding global interactions and were therefore not interpreted as consistent intervention effects.

## 4 Discussion

The main finding was that both MT and ET improved maximal muscle strength and selected cardiopulmonary performance outcomes in women with obesity, with no consistent evidence that these adaptations depended on training or testing time. In contrast, the untrained CG showed a significant increase in waist circumference. Gains in upper-limb maximal strength were significantly greater in both training groups than in CG. However, for VO_2peak_ and lower-limb maximal strength, only MT showed an overall improvement significantly greater than CG. Direct comparisons of pre-to-post changes between MT and ET were not significant. Therefore, our hypothesis that ET would promote greater adaptations than MT was not supported.

The primary effects of the combined training protocol applied in the present study were improvements in muscle strength and cardiopulmonary performance, which represent important functional adaptations in previously untrained women with obesity. These findings are clinically relevant because low muscle strength and poor cardiopulmonary fitness have been associated with an increased risk of cardiovascular events and mortality (33–35). Importantly, these adaptations were observed irrespective of whether the exercise sessions were performed in the morning or evening. Although one isolated pairwise comparison suggested greater post-intervention lower-limb strength in the MT under morning testing conditions, the absence of a significant group × period × testing-time interaction indicates that this finding should be interpreted cautiously. Overall, the pattern of results does not support a consistent time-of-day advantage for either training schedule.

The findings of the present study are consistent with Grgic et al. (36), who analyzed the effects of morning vs. evening resistance training on muscular strength and hypertrophy and reported no consistent evidence favoring one training time over another. However, acute performance may be affected when training and testing occur at the same time of day. Similarly, a recent meta-analysis investigating the influence of exercise timing on training-induced adaptations (13), specifically investigating whether the time of day of strength and endurance training influenced health- and performance-related outcomes, found little evidence that training at a specific time of day consistently produces superior adaptations.

Regarding anthropometric and body composition outcomes, no significant changes were observed following eight weeks of combined training, irrespective of the time of day at which exercise was performed. The isolated increase in waist circumference observed in the CG should be interpreted cautiously, as it occurred in the absence of any intervention and may reflect normal biological variability rather than a true intervention-related effect. The absence of reductions in body fat and body mass may be explained, at least in part, by the lack of dietary energy restriction (37), as participants were instructed to maintain their habitual dietary patterns throughout the intervention. In addition, the duration and overall training load of the intervention may not have been sufficient to elicit meaningful changes in body composition. Although protocols involving longer intervention periods or greater training frequency, volume, and/or intensity may promote greater fat loss (38), such approaches may not be appropriate for previously untrained women with obesity, as they could negatively affect adherence to the intervention (39).

Our findings are consistent with those of Brandao et al. (5) and da Silva Rodrigues (8), who reported that either eight or fourteen weeks, respectively, of combined training improved physical performance in women with obesity without significant changes in body mass or BMI. Despite differences in intervention duration, both studies employed combined aerobic and resistance exercise performed three times per week, resulting in a weekly exercise frequency similar to the present study. Likewise Brandao et al. (5), prescribed moderate-intensity aerobic exercise combined with progressive resistance training, while da Silva Rodrigues (8) implemented a similar multicomponent training approach focused primarily on improving physical fitness rather than maximizing energy expenditure. In contrast Oh and Lee (40), observed significant reductions in body mass and body fat percentage after eight weeks of combined training in middle-aged women with obesity. Although the intervention duration was identical to that of the present study, their training program incorporated a substantially greater exercise volume and energy expenditure, consisting of five supervised sessions per week (forty sessions in total) compared with three weekly sessions (twenty-four sessions) in the present investigation. Furthermore, their aerobic training was performed at moderate-to-vigorous intensity for longer durations, thereby increasing total caloric expenditure throughout the intervention. By comparison, our protocol was designed to progressively increase training volume and intensity over eight weeks, with resistance and aerobic exercises equally distributed within each session (20–30 min per modality) and aerobic exercise prescribed between 55% and 75% of VO_2peak_. Therefore, while our intervention was sufficient to improve physical performance, the lower weekly training frequency and cumulative exercise dose may have limited its effects on body mass and adiposity. These findings suggest that differences in intervention characteristics, such as exercise frequency, training volume, and overall energy expenditure, may contribute to the heterogeneity of body composition responses across studies.

The present findings indicate that, under conditions in which exercise is performed without concomitant dietary restriction, exercise timing does not appear to influence adiposity-related adaptations. Future studies should investigate whether combining exercise with caloric restriction and/or implementing protocols with greater energy expenditure modifies the effects of exercise timing on fat loss. Similarly, no changes in lean mass were observed following the intervention. This finding suggests that the training stimulus, although sufficient to improve muscle strength, may not have provided an adequate anabolic stimulus to induce measurable increases in skeletal muscle mass. Factors such as the relatively short intervention period and the absence of nutritional support may have contributed to the lack of hypertrophic adaptations. Therefore, caution is warranted when interpreting the present findings, as the absence of changes in lean mass precludes definitive conclusions regarding the influence of exercise timing on hypertrophic adaptations.

In the present study, cardiovascular responses were not consistently influenced by training time. HR, SBP, and DBP differed significantly between resting and immediately post-CPET measurements, reflecting the expected acute cardiovascular response to incremental exercise. However, these responses were broadly similar across groups, and no consistent pre-to-post changes were identified according to training group, testing time, or measurement moment. This pattern is consistent with the cardiovascular responses typically observed during exercise stress testing, in which SBP progressively increases with exercise intensity (41). The absence of significant higher-order interactions suggests that the eight-week intervention did not induce consistent chronic adaptations in the cardiovascular variables assessed. This may partly reflect the normal cardiovascular values presented by participants at baseline, which may have limited the potential for additional measurable improvement (42). Furthermore, BP was measured immediately after maximal CPET using the auscultatory method. Although this procedure follows established exercise-testing recommendations, post-exercise auscultatory measurements are inherently more variable than resting measurements because tachycardia, increased pulse pressure, and rapid hemodynamic recovery may reduce the reliability of Korotkoff sound detection. Therefore, isolated post-exercise BP findings should be interpreted with caution.

This study has some limitations that should be considered. First, the sample consisted exclusively of previously untrained women with obesity, which limits the generalizability of the findings to other populations. Second, the absence of long-term follow-up precluded the assessment of the sustainability of the observed adaptations over time. Another limitation is the substantial participant attrition observed throughout the recruitment and intervention process. Although a large number of women initially expressed interest in participating, eligibility criteria, participant availability, and losses during follow-up resulted in a considerably smaller final analytical sample. Most withdrawals were related to difficulties attending the scheduled training sessions because of work, family, or personal routine constraints rather than intervention-related adverse events. Nevertheless, this attrition may have introduced selection bias by favoring participants with greater schedule flexibility and adherence to the intervention protocol, thereby limiting the ecological validity of the findings.

Despite these losses, participant retention after initiation of the intervention was consistent with supervised exercise trials involving women with obesity, and the final sample remained sufficient to achieve the statistical power estimated a posteriori. Finally, although the initial study design considered circadian preference, the small number of participants with evening chronotype and participant losses during follow-up prevented stratified analyses according to chronotype. This is particularly relevant because circadian regulation influences glucose, lipid, and energy metabolism (43), suggesting that individual circadian characteristics should be considered in future studies investigating exercise timing. Furthermore, circadian biomarkers were not assessed, limiting a more detailed understanding of the physiological mechanisms underlying responses to MT and ET. Human skeletal muscle exhibits day-night variation in oxidative capacity, suggesting that muscle metabolism is not constant throughout the 24 h cycle (44).

Although dietary intake, sleep behavior, and habitual physical activity were monitored during the intervention, these variables were collected primarily for monitoring purposes and were not included as outcomes or covariates in the main analyses. Therefore, the absence of quantitative reporting of these lifestyle factors limits our ability to completely exclude residual confounding related to changes in diet, sleep, or daily activity patterns. Future studies should include larger sample sizes, longer intervention periods, chronotype assessment, sleep behavior, menstrual cycle phase, dietary control, and molecular or biochemical markers of circadian regulation. These approaches may help clarify whether exercise timing exerts a greater influence on metabolic and molecular responses than on functional adaptations in women with obesity.

## 5 Conclusion

Combined training performed either in the morning or evening improved maximal muscle strength and selected cardiopulmonary performance outcomes in women with obesity, while body-composition changes were limited and no consistent chronic cardiovascular adaptations were observed. Direct comparisons between MT and ET did not demonstrate significant differences in pre-to-post intervention adaptations, providing no consistent evidence of superiority according to training time. Therefore, exercise timing may be selected according to individual preferences and daily routines to support adherence without substantially compromising the functional benefits of combined training.

## Data Availability

The original contributions presented in the study are included in the article/Supplementary Material, further inquiries can be directed to the corresponding author.
