# Supplementary material for: Morning or evening? Time of day does not affect body composition, cardiopulmonary performance, and muscle strength responses to combined training in women with obesity
Source: Front Sports Act Living. 2026 Sep 15;8:1914784. doi: 10.3389/fspor.2026.1914784 (PMC13619898; doi:10.3389/fspor.2026.1914784)
Supplement: Supplementary file 1 [file Datasheet1.pdf]

Supplementary Material 1

Mixed-Effects Modeling Approach

All statistical analyses were performed using R software (R Core Team, 2025; R Foundation for Statistical Computing, Vienna, Austria) within the RStudio integrated development environment. Data management and statistical analyses were conducted using the readxl, tidyverse, janitor, lme4, lmerTest, and emmeans packages. Linear mixed-effects models were fitted using the *lmer()* function to account for the repeated-measures structure of the data and the correlation among observations obtained from the same participant. Participant identification was included as a random intercept in all models.

Fixed factors and levels

For longitudinal anthropometric and body-composition outcomes assessed before and after the intervention, the following model was fitted:

Outcome ~ Group × Experimental Period + (1 | Subject)

For physical-performance outcomes assessed during morning and evening testing sessions, the following model was fitted:

Outcome ~ Group × Experimental Period × Time of Testing + (1 | Subject)

Where the *Time of Testing* corresponded to morning or evening assessments.

For cardiovascular outcomes, a four-way interaction model was fitted:

Outcome ~ Group × Experimental Period × Testing Time + (1 | Subject)

For cardiovascular outcomes, which were assessed at rest and immediately after exercise during morning and evening testing sessions, the following model was fitted:

Outcome ~ Group × Experimental Period × Testing Time × Measurement Moment + (1 | Subject)

All predictors were treated as categorical fixed factors with predefined levels. Models were fitted in the original measurement units using restricted maximum likelihood estimation. Missing observations were not imputed. Instead, models used all available observations from participants included in the per-protocol analytical sample. Consequently, a participant with a missing value at one assessment could still contribute other available observations to the model. This approach relies on the assumption that missing observations were missing at random, conditional on the variables included in the model.

Global fixed effects were evaluated using Type III F-tests with Satterthwaite-approximated degrees of freedom. Estimated marginal means and model-based contrasts were obtained using the *emmeans* package with Kenward–Roger degrees of freedom. Pre-to-post contrasts were estimated separately within each group and, when applicable, within each testing time and measurement moment. Between-group pairwise comparisons within each experimental period and testing condition were adjusted using Tukey's method. Interaction contrasts comparing pre-to-post changes among groups were adjusted using Sidak's method. No additional multiplicity adjustment was applied to single pre-to-post or morning-versus-evening contrasts estimated separately within each group and condition.

Baseline between-group comparisons were obtained by estimating group contrasts at the pre-intervention level, separately for morning and evening testing when applicable, with Tukey adjustment. Descriptive values are reported as observed means and standard deviations, whereas inferential estimates and p values were obtained from the fitted models.

Standardized effect sizes were calculated from model-estimated pre-to-post contrasts using the *eff_size()* function. Cohen's d was obtained by dividing the estimated contrast by the residual standard deviation of the corresponding fitted model. Statistical significance was set at $p < 0.05$.

RStudio Packages and Functions

readxl (Wickham and Bryan, 2019) Imports .xls and .xlsx files into R without requiring external dependencies and returns the imported data as tibbles.

tidyverse (Wickham et al., 2019) A collection of packages designed for data science, sharing a common philosophy and syntax. Covers data import, tidying, transformation, visualization, and programming (including ggplot2, dplyr, tidyr, etc.).

janitor (Firke, 2023) Provides utilities for cleaning and examining data, including standardizing column names (snake_case), removing empty rows/columns, and generating frequency tables (tabyl).

lme4 (Bates et al., 2015) Fits linear and generalized linear mixed-effects models (LMM/GLMM), handling random effects structures for repeated measures and nested/hierarchical data.

lmerTest (Kuznetsova et al., 2017) Extends the lme4 package by providing p-values for fixed effects in linear mixed-effects models, using Satterthwaite's or Kenward-Roger's degrees of freedom approximations, essential for statistical inference in mixed models.

emmeans (Lenth, 2024) Computes estimated marginal means and supports model-based pairwise comparisons, interaction contrasts, and multiple-comparison adjustments. In the present study, it was used to estimate pre-to-post and between-group contrasts, apply Tukey or Sidak adjustments when appropriate, and calculate standardized effect sizes using the eff_size() function.

References

Wickham, H., and Bryan, J. (2019). readxl: Read Excel Files. Available at: https://CRAN.R-project.org/package=readxl

Wickham, H., Averick, M., Bryan, J., Chang, W., McGowan, L. D., François, R., et al. (2019). Welcome to the Tidyverse. J. Open Source Softw. 4, 1686. doi:10.21105/joss.01686

Firke, S. (2023). janitor: Simple Tools for Examining and Cleaning Dirty Data. Available at: https://CRAN.R-project.org/package=janitor

Bates, D., Mächler, M., Bolker, B., and Walker, S. (2015). Fitting linear mixed-effects models using lme4. J. Stat. Softw. 67, 1–48. doi:10.18637/jss.v067.i01

Kuznetsova, A., Brockhoff, P. B., and Christensen, R. H. B. (2017). lmerTest package: tests in linear mixed effects models. J. Stat. Softw. 82, 1–26. doi:10.18637/jss.v082.i13

Lenth, R. V. (2024). emmeans: Estimated Marginal Means, aka Least-Squares Means. Available at: https://CRAN.R-project.org/package=emmeans

RStudio Packages and Functions

```
##################################################################################
# STATISTICAL ANALYSIS
# Effects of Morning vs. Evening Combined Training
################################################################################################
#
# 1. PACKAGE SETUP ---------------------------------------------------------------------------
# Install packages manually before running the analysis, if necessary:
# install.packages(c(
#   "readxl",
#   "tidyverse",
#   "janitor",
#   "lme4",
#   "lmerTest",
#   "emmeans"
# ))
library(readxl)
library(tidyverse)
library(janitor)
library(lme4)
library(lmerTest)
library(emmeans)
# 2. GLOBAL CONTRAST SETTINGS -------------------------------------------------------------
options(
  contrasts = c(
    "contr.sum",
    "contr.poly"
  )
)
# 3. DATA IMPORT -----------------------------------------------------------------------------
data_file <- "submission_data_english.xlsx"
participant_data <- readxl::read_excel(
  path = data_file,
  sheet = "participant_ids"
) |>
  janitor::clean_names()
assessment_data <- readxl::read_excel(
  path = data_file,
  sheet = "assessments"
) |>
  janitor::clean_names()
cardio_data <- readxl::read_excel(
  path = data_file,
  sheet = "cardio"
) |>
```

```r
  janitor::clean_names()
strength_data <- readxl::read_excel(
  path = data_file,
  sheet = "strength"
) |>
  janitor::clean_names()
# 4. INITIAL DATA CHECKS -----------------------------------------------------
glimpse(participant_data)
glimpse(assessment_data)
glimpse(cardio_data)
glimpse(strength_data)


################################################################################
#
# MODEL 1.1: ANTHROPOMETRY — BODY MASS
################################################################################
#
# 1. DATA PREPARATION --------------------------------------------------------
body_mass_data <- assessment_data |>
  select(
    id,
    group,
    experimental_period,
    body_mass_kg
  ) |>
  mutate(
    id = factor(id),
    group = factor(
      group,
      levels = c("CG", "MT", "ET")
    ),
    experimental_period = factor(
      experimental_period,
      levels = c("pre", "post")
    )
  )
stopifnot(is.numeric(body_mass_data$body_mass_kg))
# 2. DESCRIPTIVE STATISTICS --------------------------------------------------
body_mass_descriptive <- body_mass_data |>
  group_by(
    group,
    experimental_period
  ) |>
  summarise(
    n = sum(!is.na(body_mass_kg)),
```

```
    mean = mean(body_mass_kg, na.rm = TRUE),
    standard_deviation = sd(body_mass_kg, na.rm = TRUE),
    median = median(body_mass_kg, na.rm = TRUE),
    minimum = min(body_mass_kg, na.rm = TRUE),
    maximum = max(body_mass_kg, na.rm = TRUE),
    missing_values = sum(is.na(body_mass_kg)),
    .groups = "drop"
  )
body_mass_descriptive
# 3. LINEAR MIXED-EFFECTS MODEL ---------------------------------------------------
body_mass_model <- lmer(
  body_mass_kg ~ group * experimental_period + (1 | id),
  data = body_mass_data,
  REML = TRUE,
  na.action = na.exclude
)
summary(body_mass_model)
# 4. GLOBAL FIXED-EFFECT TESTS ----------------------------------------------------
body_mass_anova <- anova(
  body_mass_model,
  type = 3,
  ddf = "Satterthwaite"
)
body_mass_anova
# 5. ESTIMATED MARGINAL MEANS -------------------------------------------------------

body_mass_emmeans <- emmeans(
  body_mass_model,
  ~ group * experimental_period
)
body_mass_emmeans
# 6. WITHIN-GROUP PRE–POST CONTRASTS ----------------------------------------------
body_mass_period_contrasts <- contrast(
  body_mass_emmeans,
  method = "revpairwise",
  by = "group",
  adjust = "none"
)
summary(
  body_mass_period_contrasts,
  infer = c(TRUE, TRUE)
)
# 7. BETWEEN-GROUP CONTRASTS AT EACH PERIOD ----------------------------------
body_mass_group_contrasts <- contrast(
  body_mass_emmeans,
  method = "pairwise",
```

```
  by = "experimental_period",
  adjust = "tukey"
)
summary(
  body_mass_group_contrasts,
  infer = c(TRUE, TRUE)
)
# 8. WITHIN-GROUP STANDARDIZED EFFECT SIZES -----------------------------------

body_mass_period_emmeans <- emmeans(
  body_mass_model,
  ~ experimental_period | group
)
body_mass_effect_sizes <- eff_size(
  body_mass_period_emmeans,
  sigma = sigma(body_mass_model),
  edf = df.residual(body_mass_model),
  method = "revpairwise"
)
body_mass_effect_sizes
# 9. ANALYTICAL SAMPLE -------------------------------------------------------
nobs(body_mass_model)
nlevels(body_mass_data$id)
###############################################################################
#
# MODEL 1.2: ANTHROPOMETRY — BODY MASS INDEX
###############################################################################
#
# 1. DATA PREPARATION --------------------------------------------------------
bmi_data <- assessment_data |>
  select(
    id,
    group,
    experimental_period,
    bmi
  ) |>
  mutate(
    id = factor(id),
    group = factor(
      group,
      levels = c("CG", "MT", "ET")
    ),
    experimental_period = factor(
      experimental_period,
      levels = c("pre", "post")
    )
```

```
  )
stopifnot(is.numeric(bmi_data$bmi))
# 2. DESCRIPTIVE STATISTICS ---------------------------------------------------
bmi_descriptive <- bmi_data |>
  group_by(
    group,
    experimental_period
  ) |>
  summarise(
    n = sum(!is.na(bmi)),
    mean = mean(bmi, na.rm = TRUE),
    standard_deviation = sd(bmi, na.rm = TRUE),
    median = median(bmi, na.rm = TRUE),
    missing_values = sum(is.na(bmi)),
    .groups = "drop"
  )
print(
  bmi_descriptive,
  width = Inf,
  n = Inf
)
# 3. LINEAR MIXED-EFFECTS MODEL ------------------------------------------------
bmi_model <- lmer(
  bmi ~ group * experimental_period + (1 | id),
  data = bmi_data,
  REML = TRUE,
  na.action = na.exclude
)
summary(bmi_model)
# 4. GLOBAL FIXED-EFFECT TESTS -------------------------------------------------
bmi_anova <- anova(
  bmi_model,
  type = 3,
  ddf = "Satterthwaite"
)
bmi_anova
# 5. ESTIMATED MARGINAL MEANS --------------------------------------------------
bmi_emmeans <- emmeans(
  bmi_model,
  ~ group * experimental_period
)
bmi_emmeans
# 6. WITHIN-GROUP PRE–POST CONTRASTS -------------------------------------------
bmi_period_contrasts <- contrast(
  bmi_emmeans,
  method = "revpairwise",
```

```r
  by = "group",
  adjust = "none"
)
summary(
  bmi_period_contrasts,
  infer = c(TRUE, TRUE)
)
# 7. BETWEEN-GROUP CONTRASTS AT EACH PERIOD -----------------------------------
bmi_group_contrasts <- contrast(
  bmi_emmeans,
  method = "pairwise",
  by = "experimental_period",
  adjust = "tukey"
)
summary(
  bmi_group_contrasts,
  infer = c(TRUE, TRUE)
)
# 8. WITHIN-GROUP STANDARDIZED EFFECT SIZES -----------------------------------
bmi_period_emmeans <- emmeans(
  bmi_model,
  ~ experimental_period | group
)
bmi_effect_sizes <- eff_size(
  bmi_period_emmeans,
  sigma = sigma(bmi_model),
  edf = df.residual(bmi_model),
  method = "revpairwise"
)
bmi_effect_sizes
# 9. ANALYTICAL SAMPLE --------------------------------------------------------

nobs(bmi_model)
nlevels(droplevels(bmi_data[!is.na(bmi_data$bmi), ])$id)
##############################################################################
#
# MODEL 1.3: ANTHROPOMETRY — WAIST CIRCUMFERENCE
##############################################################################
#
# 1. DATA PREPARATION ---------------------------------------------------------
waist_circumference_data <- assessment_data |>
  select(
    id,
    group,
    experimental_period,
    waist_circumference_cm
```

```
  ) |>
  mutate(
    id = factor(id),
    group = factor(
      group,
      levels = c("CG", "MT", "ET")
    ),
    experimental_period = factor(
      experimental_period,
      levels = c("pre", "post")
    )
  )
stopifnot(is.numeric(waist_circumference_data$waist_circumference_cm))
# 2. DESCRIPTIVE STATISTICS --------------------------------------------------
waist_circumference_descriptive <- waist_circumference_data |>
  group_by(
    group,
    experimental_period
  ) |>
  summarise(
    n = sum(!is.na(waist_circumference_cm)),
    mean = mean(waist_circumference_cm, na.rm = TRUE),
    standard_deviation = sd(waist_circumference_cm, na.rm = TRUE),
    median = median(waist_circumference_cm, na.rm = TRUE),
    missing_values = sum(is.na(waist_circumference_cm)),
    .groups = "drop"
  )
print(
  waist_circumference_descriptive,
  width = Inf,
  n = Inf
)
# 3. LINEAR MIXED-EFFECTS MODEL ----------------------------------------------
waist_circumference_model <- lmer(
  waist_circumference_cm ~ group * experimental_period + (1 | id),
  data = waist_circumference_data,
  REML = TRUE,
  na.action = na.exclude
)
summary(waist_circumference_model)
# 4. GLOBAL FIXED-EFFECT TESTS -----------------------------------------------
waist_circumference_anova <- anova(
  waist_circumference_model,
  type = 3,
  ddf = "Satterthwaite"
)
```

```
waist_circumference_anova
# 5. ESTIMATED MARGINAL MEANS -----------------------------------------------
waist_circumference_emmeans <- emmeans(
  waist_circumference_model,
  ~ group * experimental_period
)
waist_circumference_emmeans
# 6. WITHIN-GROUP PRE–POST CONTRASTS ------------------------------------------
waist_circumference_period_contrasts <- contrast(
  waist_circumference_emmeans,
  method = "revpairwise",
  by = "group",
  adjust = "none"
)
summary(
  waist_circumference_period_contrasts,
  infer = c(TRUE, TRUE)
)
# 7. BETWEEN-GROUP CONTRASTS AT EACH PERIOD ----------------------------------
waist_circumference_group_contrasts <- contrast(
  waist_circumference_emmeans,
  method = "pairwise",
  by = "experimental_period",
  adjust = "tukey"
)
summary(
  waist_circumference_group_contrasts,
  infer = c(TRUE, TRUE)
)
# 8. WITHIN-GROUP STANDARDIZED EFFECT SIZES ----------------------------------
waist_circumference_period_emmeans <- emmeans(
  waist_circumference_model,
  ~ experimental_period | group
)
waist_circumference_effect_sizes <- eff_size(
  waist_circumference_period_emmeans,
  sigma = sigma(waist_circumference_model),
  edf = df.residual(waist_circumference_model),
  method = "revpairwise"
)
waist_circumference_effect_sizes
# 9. ANALYTICAL SAMPLE -------------------------------------------------------
nobs(waist_circumference_model)
nlevels(
  droplevels(
    waist_circumference_data[
```

```
      !is.na(waist_circumference_data$waist_circumference_cm),
    ]
  )$id
)
##################################################################################
#
# MODEL 1.4: ANTHROPOMETRY — HIP CIRCUMFERENCE
##################################################################################
#
# 1. DATA PREPARATION ----------------------------------------------------------

hip_circumference_data <- assessment_data |>
  select(
    id,
    group,
    experimental_period,
    hip_circumference_cm
  ) |>
  mutate(
    id = factor(id),
    group = factor(
      group,
      levels = c("CG", "MT", "ET")
    ),
    experimental_period = factor(
      experimental_period,
      levels = c("pre", "post")
    )
  )
stopifnot(is.numeric(hip_circumference_data$hip_circumference_cm))
# 2. DESCRIPTIVE STATISTICS ----------------------------------------------------
hip_circumference_descriptive <- hip_circumference_data |>
  group_by(
    group,
    experimental_period
  ) |>
  summarise(
    n = sum(!is.na(hip_circumference_cm)),
    mean = mean(hip_circumference_cm, na.rm = TRUE),
    standard_deviation = sd(hip_circumference_cm, na.rm = TRUE),
    median = median(hip_circumference_cm, na.rm = TRUE),
    missing_values = sum(is.na(hip_circumference_cm)),
    .groups = "drop"
  )
print(
  hip_circumference_descriptive,
```

```r
  width = Inf,
  n = Inf
)
# 3. LINEAR MIXED-EFFECTS MODEL ------------------------------------------------
hip_circumference_model <- lmer(
  hip_circumference_cm ~ group * experimental_period + (1 | id),
  data = hip_circumference_data,
  REML = TRUE,
  na.action = na.exclude
)
summary(hip_circumference_model)
# 4. GLOBAL FIXED-EFFECT TESTS ------------------------------------------------
hip_circumference_anova <- anova(
  hip_circumference_model,
  type = 3,
  ddf = "Satterthwaite"
)
hip_circumference_anova
# 5. ESTIMATED MARGINAL MEANS -------------------------------------------------
hip_circumference_emmeans <- emmeans(
  hip_circumference_model,
  ~ group * experimental_period
)
hip_circumference_emmeans
# 6. WITHIN-GROUP PRE–POST CONTRASTS ------------------------------------------
hip_circumference_period_contrasts <- contrast(
  hip_circumference_emmeans,
  method = "revpairwise",
  by = "group",
  adjust = "none"
)
summary(
  hip_circumference_period_contrasts,
  infer = c(TRUE, TRUE)
)
# 7. BETWEEN-GROUP CONTRASTS AT EACH PERIOD ----------------------------------
hip_circumference_group_contrasts <- contrast(
  hip_circumference_emmeans,
  method = "pairwise",
  by = "experimental_period",
  adjust = "tukey"
)
summary(
  hip_circumference_group_contrasts,
  infer = c(TRUE, TRUE)
)
```

```r
# 8. WITHIN-GROUP STANDARDIZED EFFECT SIZES -----------------------------------
hip_circumference_period_emmeans <- emmeans(
  hip_circumference_model,
  ~ experimental_period | group
)
hip_circumference_effect_sizes <- eff_size(
  hip_circumference_period_emmeans,
  sigma = sigma(hip_circumference_model),
  edf = df.residual(hip_circumference_model),
  method = "revpairwise"
)
hip_circumference_effect_sizes
# 9. ANALYTICAL SAMPLE -------------------------------------------------------
nobs(hip_circumference_model)
nlevels(
  droplevels(
    hip_circumference_data[
      !is.na(hip_circumference_data$hip_circumference_cm),
    ]
  )$id
)
################################################################################
#
# MODEL 1.5: BODY COMPOSITION — BODY FAT PERCENTAGE
################################################################################
#
# 1. DATA PREPARATION -------------------------------------------------------
body_fat_percentage_data <- assessment_data |>
  select(
    id,
    group,
    experimental_period,
    body_fat_percentage
  ) |>
  mutate(
    id = factor(id),
    group = factor(
      group,
      levels = c("CG", "MT", "ET")
    ),
    experimental_period = factor(
      experimental_period,
      levels = c("pre", "post")
    )
  )
stopifnot(is.numeric(body_fat_percentage_data$body_fat_percentage))
```

```
# 2. DESCRIPTIVE STATISTICS ---------------------------------------------------
body_fat_percentage_descriptive <- body_fat_percentage_data |>
  group_by(
    group,
    experimental_period
  ) |>
  summarise(
    n = sum(!is.na(body_fat_percentage)),
    mean = mean(body_fat_percentage, na.rm = TRUE),
    standard_deviation = sd(body_fat_percentage, na.rm = TRUE),
    median = median(body_fat_percentage, na.rm = TRUE),
    missing_values = sum(is.na(body_fat_percentage)),
    .groups = "drop"
  )
print(
  body_fat_percentage_descriptive,
  width = Inf,
  n = Inf
)
# 3. LINEAR MIXED-EFFECTS MODEL -----------------------------------------------
body_fat_percentage_model <- lmer(
  body_fat_percentage ~ group * experimental_period + (1 | id),
  data = body_fat_percentage_data,
  REML = TRUE,
  na.action = na.exclude
)
summary(body_fat_percentage_model)
# 4. GLOBAL FIXED-EFFECT TESTS ------------------------------------------------
body_fat_percentage_anova <- anova(
  body_fat_percentage_model,
  type = 3,
  ddf = "Satterthwaite"
)
body_fat_percentage_anova
# 5. ESTIMATED MARGINAL MEANS -------------------------------------------------
body_fat_percentage_emmeans <- emmeans(
  body_fat_percentage_model,
  ~ group * experimental_period
)
body_fat_percentage_emmeans
# 6. WITHIN-GROUP PRE–POST CONTRASTS ------------------------------------------
body_fat_percentage_period_contrasts <- contrast(
  body_fat_percentage_emmeans,
  method = "revpairwise",
  by = "group",
  adjust = "none"
```

```
)
summary(
  body_fat_percentage_period_contrasts,
  infer = c(TRUE, TRUE)
)
# 7. BETWEEN-GROUP CONTRASTS AT EACH PERIOD ----------------------------------
body_fat_percentage_group_contrasts <- contrast(
  body_fat_percentage_emmeans,
  method = "pairwise",
  by = "experimental_period",
  adjust = "tukey"
)
summary(
  body_fat_percentage_group_contrasts,
  infer = c(TRUE, TRUE)
)
# 8. WITHIN-GROUP STANDARDIZED EFFECT SIZES ----------------------------------
body_fat_percentage_period_emmeans <- emmeans(
  body_fat_percentage_model,
  ~ experimental_period | group
)
body_fat_percentage_effect_sizes <- eff_size(
  body_fat_percentage_period_emmeans,
  sigma = sigma(body_fat_percentage_model),
  edf = df.residual(body_fat_percentage_model),
  method = "revpairwise"
)
body_fat_percentage_effect_sizes
# 9. ANALYTICAL SAMPLE -------------------------------------------------------
nobs(body_fat_percentage_model)
nlevels(
  droplevels(
    body_fat_percentage_data[
      !is.na(body_fat_percentage_data$body_fat_percentage),
    ]
  )$id
)
###############################################################################
#
# MODEL 1.6: BODY COMPOSITION — LEAN MASS
###############################################################################
#
# 1. DATA PREPARATION --------------------------------------------------------
lean_mass_data <- assessment_data |>
  select(
    id,
```

```r
    group,
    experimental_period,
    lean_mass_kg
  ) |>
  mutate(
    id = factor(id),
    group = factor(
      group,
      levels = c("CG", "MT", "ET")
    ),
    experimental_period = factor(
      experimental_period,
      levels = c("pre", "post")
    )
  )
stopifnot(is.numeric(lean_mass_data$lean_mass_kg))
# 2. DESCRIPTIVE STATISTICS ---------------------------------------------------
lean_mass_descriptive <- lean_mass_data |>
  group_by(
    group,
    experimental_period
  ) |>
  summarise(
    n = sum(!is.na(lean_mass_kg)),
    mean = mean(lean_mass_kg, na.rm = TRUE),
    standard_deviation = sd(lean_mass_kg, na.rm = TRUE),
    median = median(lean_mass_kg, na.rm = TRUE),
    missing_values = sum(is.na(lean_mass_kg)),
    .groups = "drop"
  )
print(
  lean_mass_descriptive,
  width = Inf,
  n = Inf
)
# 3. LINEAR MIXED-EFFECTS MODEL ----------------------------------------------
lean_mass_model <- lmer(
  lean_mass_kg ~ group * experimental_period + (1 | id),
  data = lean_mass_data,
  REML = TRUE,
  na.action = na.exclude
)
summary(lean_mass_model)
# 4. GLOBAL FIXED-EFFECT TESTS -----------------------------------------------
lean_mass_anova <- anova(
  lean_mass_model,
```

```
  type = 3,
  ddf = "Satterthwaite"
)
lean_mass_anova
# 5. ESTIMATED MARGINAL MEANS --------------------------------------------------
lean_mass_emmeans <- emmeans(
  lean_mass_model,
  ~ group * experimental_period
)
lean_mass_emmeans
# 6. WITHIN-GROUP PRE–POST CONTRASTS ---------------------------------------------
lean_mass_period_contrasts <- contrast(
  lean_mass_emmeans,
  method = "revpairwise",
  by = "group",
  adjust = "none"
)
summary(
  lean_mass_period_contrasts,
  infer = c(TRUE, TRUE)
)
# 7. BETWEEN-GROUP CONTRASTS AT EACH PERIOD ------------------------------------
lean_mass_group_contrasts <- contrast(
  lean_mass_emmeans,
  method = "pairwise",
  by = "experimental_period",
  adjust = "tukey"
)
summary(
  lean_mass_group_contrasts,
  infer = c(TRUE, TRUE)
)
# 8. WITHIN-GROUP STANDARDIZED EFFECT SIZES ------------------------------------
lean_mass_period_emmeans <- emmeans(
  lean_mass_model,
  ~ experimental_period | group
)
lean_mass_effect_sizes <- eff_size(
  lean_mass_period_emmeans,
  sigma = sigma(lean_mass_model),
  edf = df.residual(lean_mass_model),
  method = "revpairwise"
)
lean_mass_effect_sizes
# 9. ANALYTICAL SAMPLE -------------------------------------------------------
nobs(lean_mass_model)
```

```r
nlevels(
  droplevels(
    lean_mass_data[
      !is.na(lean_mass_data$lean_mass_kg),
    ]
  )$id
)
#############################################################################################
#
# MODEL 1.7: BODY COMPOSITION — FAT MASS
#############################################################################################
#
# 1. DATA PREPARATION ---------------------------------------------------------
fat_mass_data <- assessment_data |>
  select(
    id,
    group,
    experimental_period,
    fat_mass_kg
  ) |>
  mutate(
    id = factor(id),
    group = factor(
      group,
      levels = c("CG", "MT", "ET")
    ),
    experimental_period = factor(
      experimental_period,
      levels = c("pre", "post")
    )
  )
stopifnot(is.numeric(fat_mass_data$fat_mass_kg))
# 2. DESCRIPTIVE STATISTICS ---------------------------------------------------
fat_mass_descriptive <- fat_mass_data |>
  group_by(
    group,
    experimental_period
  ) |>
  summarise(
    n = sum(!is.na(fat_mass_kg)),
    mean = mean(fat_mass_kg, na.rm = TRUE),
    standard_deviation = sd(fat_mass_kg, na.rm = TRUE),
    median = median(fat_mass_kg, na.rm = TRUE),
    missing_values = sum(is.na(fat_mass_kg)),
    .groups = "drop"
  )
```

```r
print(
  fat_mass_descriptive,
  width = Inf,
  n = Inf
)
# 3. LINEAR MIXED-EFFECTS MODEL -----------------------------------------------------
fat_mass_model <- lmer(
  fat_mass_kg ~ group * experimental_period + (1 | id),
  data = fat_mass_data,
  REML = TRUE,
  na.action = na.exclude
)
summary(fat_mass_model)
# 4. GLOBAL FIXED-EFFECT TESTS ------------------------------------------------------
fat_mass_anova <- anova(
  fat_mass_model,
  type = 3,
  ddf = "Satterthwaite"
)
fat_mass_anova
# 5. ESTIMATED MARGINAL MEANS -------------------------------------------------------
fat_mass_emmeans <- emmeans(
  fat_mass_model,
  ~ group * experimental_period
)
fat_mass_emmeans
# 6. WITHIN-GROUP PRE–POST CONTRASTS ------------------------------------------------
fat_mass_period_contrasts <- contrast(
  fat_mass_emmeans,
  method = "revpairwise",
  by = "group",
  adjust = "none"
)
summary(
  fat_mass_period_contrasts,
  infer = c(TRUE, TRUE)
)
# 7. BETWEEN-GROUP CONTRASTS AT EACH PERIOD -----------------------------------------
fat_mass_group_contrasts <- contrast(
  fat_mass_emmeans,
  method = "pairwise",
  by = "experimental_period",
  adjust = "tukey"
)
summary(
  fat_mass_group_contrasts,
```

```
  infer = c(TRUE, TRUE)
)
# 8. WITHIN-GROUP STANDARDIZED EFFECT SIZES -----------------------------------
fat_mass_period_emmeans <- emmeans(
  fat_mass_model,
  ~ experimental_period | group
)
fat_mass_effect_sizes <- eff_size(
  fat_mass_period_emmeans,
  sigma = sigma(fat_mass_model),
  edf = df.residual(fat_mass_model),
  method = "revpairwise"
)
fat_mass_effect_sizes
# 9. ANALYTICAL SAMPLE --------------------------------------------------------
nobs(fat_mass_model)
nlevels(
  droplevels(
    fat_mass_data[
      !is.na(fat_mass_data$fat_mass_kg),
    ]
  )$id
)
###############################################################################
#
# MODEL 1.8: BODY COMPOSITION — FAT-FREE MASS
###############################################################################
#


# 1. DATA PREPARATION ---------------------------------------------------------
fat_free_mass_data <- assessment_data |>
  select(
    id,
    group,
    experimental_period,
    fat_free_mass_kg
  ) |>
  mutate(
    id = factor(id),
    group = factor(
      group,
      levels = c("CG", "MT", "ET")
    ),
    experimental_period = factor(
      experimental_period,
```

```
      levels = c("pre", "post")
    )
  )
stopifnot(is.numeric(fat_free_mass_data$fat_free_mass_kg))
# 2. DESCRIPTIVE STATISTICS ---------------------------------------------------
fat_free_mass_descriptive <- fat_free_mass_data |>
  group_by(
    group,
    experimental_period
  ) |>
  summarise(
    n = sum(!is.na(fat_free_mass_kg)),
    mean = mean(fat_free_mass_kg, na.rm = TRUE),
    standard_deviation = sd(fat_free_mass_kg, na.rm = TRUE),
    median = median(fat_free_mass_kg, na.rm = TRUE),
    missing_values = sum(is.na(fat_free_mass_kg)),
    .groups = "drop"
  )
print(
  fat_free_mass_descriptive,
  width = Inf,
  n = Inf
)
# 3. LINEAR MIXED-EFFECTS MODEL ------------------------------------------------
fat_free_mass_model <- lmer(
  fat_free_mass_kg ~ group * experimental_period + (1 | id),
  data = fat_free_mass_data,
  REML = TRUE,
  na.action = na.exclude
)
summary(fat_free_mass_model)
# 4. GLOBAL FIXED-EFFECT TESTS -------------------------------------------------
fat_free_mass_anova <- anova(
  fat_free_mass_model,
  type = 3,
  ddf = "Satterthwaite"
)
fat_free_mass_anova
# 5. ESTIMATED MARGINAL MEANS --------------------------------------------------
fat_free_mass_emmeans <- emmeans(
  fat_free_mass_model,
  ~ group * experimental_period
)
fat_free_mass_emmeans
# 6. WITHIN-GROUP PRE–POST CONTRASTS ------------------------------------------
fat_free_mass_period_contrasts <- contrast(
```

```
  fat_free_mass_emmeans,
  method = "revpairwise",
  by = "group",
  adjust = "none"
)
summary(
  fat_free_mass_period_contrasts,
  infer = c(TRUE, TRUE)
)
# 7. BETWEEN-GROUP CONTRASTS AT EACH PERIOD -----------------------------------
fat_free_mass_group_contrasts <- contrast(
  fat_free_mass_emmeans,
  method = "pairwise",
  by = "experimental_period",
  adjust = "tukey"
)
summary(
  fat_free_mass_group_contrasts,
  infer = c(TRUE, TRUE)
)
# 8. WITHIN-GROUP STANDARDIZED EFFECT SIZES -----------------------------------
fat_free_mass_period_emmeans <- emmeans(
  fat_free_mass_model,
  ~ experimental_period | group
)
fat_free_mass_effect_sizes <- eff_size(
  fat_free_mass_period_emmeans,
  sigma = sigma(fat_free_mass_model),
  edf = df.residual(fat_free_mass_model),
  method = "revpairwise"
)
fat_free_mass_effect_sizes
# 9. ANALYTICAL SAMPLE --------------------------------------------------------
nobs(fat_free_mass_model)
nlevels(
  droplevels(
    fat_free_mass_data[
      !is.na(fat_free_mass_data$fat_free_mass_kg),
    ]
  )$id
)
###############################################################################
#
# MODEL 1.9: BODY COMPOSITION — SKELETAL MUSCLE MASS
###############################################################################
#
```

```
# 1. DATA PREPARATION ---------------------------------------------------------
skeletal_muscle_mass_data <- assessment_data |>
  select(
    id,
    group,
    experimental_period,
    skeletal_muscle_mass_kg
  ) |>
  mutate(
    id = factor(id),
    group = factor(
      group,
      levels = c("CG", "MT", "ET")
    ),
    experimental_period = factor(
      experimental_period,
      levels = c("pre", "post")
    )
  )
stopifnot(
  is.numeric(
    skeletal_muscle_mass_data$skeletal_muscle_mass_kg
  )
)
# 2. DESCRIPTIVE STATISTICS ---------------------------------------------------
skeletal_muscle_mass_descriptive <- skeletal_muscle_mass_data |>
  group_by(
    group,
    experimental_period
  ) |>
  summarise(
    n = sum(!is.na(skeletal_muscle_mass_kg)),
    mean = mean(skeletal_muscle_mass_kg, na.rm = TRUE),
    standard_deviation = sd(
      skeletal_muscle_mass_kg,
      na.rm = TRUE
    ),
    median = median(skeletal_muscle_mass_kg, na.rm = TRUE),
    missing_values = sum(is.na(skeletal_muscle_mass_kg)),
    .groups = "drop"
  )
print(
  skeletal_muscle_mass_descriptive,
  width = Inf,
  n = Inf
)
```

```
# 3. LINEAR MIXED-EFFECTS MODEL ----------------------------------------------
skeletal_muscle_mass_model <- lmer(
  skeletal_muscle_mass_kg ~
    group * experimental_period + (1 | id),
  data = skeletal_muscle_mass_data,
  REML = TRUE,
  na.action = na.exclude
)
summary(skeletal_muscle_mass_model)
# 4. GLOBAL FIXED-EFFECT TESTS ------------------------------------------------
skeletal_muscle_mass_anova <- anova(
  skeletal_muscle_mass_model,
  type = 3,
  ddf = "Satterthwaite"
)
skeletal_muscle_mass_anova
# 5. ESTIMATED MARGINAL MEANS ------------------------------------------------
skeletal_muscle_mass_emmeans <- emmeans(
  skeletal_muscle_mass_model,
  ~ group * experimental_period
)
skeletal_muscle_mass_emmeans
# 6. WITHIN-GROUP PRE–POST CONTRASTS -----------------------------------------
skeletal_muscle_mass_period_contrasts <- contrast(
  skeletal_muscle_mass_emmeans,
  method = "revpairwise",
  by = "group",
  adjust = "none"
)
summary(
  skeletal_muscle_mass_period_contrasts,
  infer = c(TRUE, TRUE)
)
# 7. BETWEEN-GROUP CONTRASTS AT EACH PERIOD ----------------------------------
skeletal_muscle_mass_group_contrasts <- contrast(
  skeletal_muscle_mass_emmeans,
  method = "pairwise",
  by = "experimental_period",
  adjust = "tukey"
)
summary(
  skeletal_muscle_mass_group_contrasts,
  infer = c(TRUE, TRUE)
)
# 8. WITHIN-GROUP STANDARDIZED EFFECT SIZES ----------------------------------
skeletal_muscle_mass_period_emmeans <- emmeans(
```

```r
  skeletal_muscle_mass_model,
  ~ experimental_period | group
)
skeletal_muscle_mass_effect_sizes <- eff_size(
  skeletal_muscle_mass_period_emmeans,
  sigma = sigma(skeletal_muscle_mass_model),
  edf = df.residual(skeletal_muscle_mass_model),
  method = "revpairwise"
)
skeletal_muscle_mass_effect_sizes
# 9. ANALYTICAL SAMPLE ---------------------------------------------------------
nobs(skeletal_muscle_mass_model)
nlevels(
  droplevels(
    skeletal_muscle_mass_data[
      !is.na(
        skeletal_muscle_mass_data$skeletal_muscle_mass_kg
      ),
    ]
  )$id
)
################################################################################
#
# MODEL 1.10: BODY COMPOSITION — TOTAL BODY WATER
################################################################################
#
# 1. DATA PREPARATION ----------------------------------------------------------
total_body_water_data <- assessment_data |>
  select(
    id,
    group,
    experimental_period,
    total_body_water_l
  ) |>
  mutate(
    id = factor(id),
    group = factor(
      group,
      levels = c("CG", "MT", "ET")
    ),
    experimental_period = factor(
      experimental_period,
      levels = c("pre", "post")
    )
  )
stopifnot(
```

```
  is.numeric(total_body_water_data$total_body_water_l)
)
# 2. DESCRIPTIVE STATISTICS ---------------------------------------------------
total_body_water_descriptive <- total_body_water_data |>
  group_by(
    group,
    experimental_period
  ) |>
  summarise(
    n = sum(!is.na(total_body_water_l)),
    mean = mean(total_body_water_l, na.rm = TRUE),
    standard_deviation = sd(
      total_body_water_l,
      na.rm = TRUE
    ),
    median = median(total_body_water_l, na.rm = TRUE),
    missing_values = sum(is.na(total_body_water_l)),
    .groups = "drop"
  )
print(
  total_body_water_descriptive,
  width = Inf,
  n = Inf
)
# 3. LINEAR MIXED-EFFECTS MODEL -----------------------------------------------
total_body_water_model <- lmer(
  total_body_water_l ~
    group * experimental_period + (1 | id),
  data = total_body_water_data,
  REML = TRUE,
  na.action = na.exclude
)
summary(total_body_water_model)
# 4. GLOBAL FIXED-EFFECT TESTS ------------------------------------------------
total_body_water_anova <- anova(
  total_body_water_model,
  type = 3,
  ddf = "Satterthwaite"
)
total_body_water_anova
# 5. ESTIMATED MARGINAL MEANS -------------------------------------------------
total_body_water_emmeans <- emmeans(
  total_body_water_model,
  ~ group * experimental_period
)
total_body_water_emmeans
```

```
# 6. WITHIN-GROUP PRE–POST CONTRASTS ------------------------------------------
total_body_water_period_contrasts <- contrast(
  total_body_water_emmeans,
  method = "revpairwise",
  by = "group",
  adjust = "none"
)
summary(
  total_body_water_period_contrasts,
  infer = c(TRUE, TRUE)
)
# 7. BETWEEN-GROUP CONTRASTS AT EACH PERIOD -----------------------------------
total_body_water_group_contrasts <- contrast(
  total_body_water_emmeans,
  method = "pairwise",
  by = "experimental_period",
  adjust = "tukey"
)
summary(
  total_body_water_group_contrasts,
  infer = c(TRUE, TRUE)
)
# 8. WITHIN-GROUP STANDARDIZED EFFECT SIZES -----------------------------------
total_body_water_period_emmeans <- emmeans(
  total_body_water_model,
  ~ experimental_period | group
)
total_body_water_effect_sizes <- eff_size(
  total_body_water_period_emmeans,
  sigma = sigma(total_body_water_model),
  edf = df.residual(total_body_water_model),
  method = "revpairwise"
)
total_body_water_effect_sizes
# 9. ANALYTICAL SAMPLE --------------------------------------------------------
nobs(total_body_water_model)
nlevels(
  droplevels(
    total_body_water_data[
      !is.na(
        total_body_water_data$total_body_water_l
      ),
    ]
  )$id
)
```

```r
################################################################################
#
# MODEL 1.11: BODY COMPOSITION — VISCERAL FAT AREA
################################################################################
#
# 1. DATA PREPARATION --------------------------------------------------------
visceral_fat_area_data <- assessment_data |>
  select(
    id,
    group,
    experimental_period,
    visceral_fat_area_cm2
  ) |>
  mutate(
    id = factor(id),
    group = factor(
      group,
      levels = c("CG", "MT", "ET")
    ),
    experimental_period = factor(
      experimental_period,
      levels = c("pre", "post")
    )
  )
stopifnot(
  is.numeric(visceral_fat_area_data$visceral_fat_area_cm2)
)
# 2. DESCRIPTIVE STATISTICS --------------------------------------------------

visceral_fat_area_descriptive <- visceral_fat_area_data |>
  group_by(
    group,
    experimental_period
  ) |>
  summarise(
    n = sum(!is.na(visceral_fat_area_cm2)),
    mean = mean(visceral_fat_area_cm2, na.rm = TRUE),
    standard_deviation = sd(
      visceral_fat_area_cm2,
      na.rm = TRUE
    ),
    median = median(
      visceral_fat_area_cm2,
      na.rm = TRUE
    ),
    missing_values = sum(is.na(visceral_fat_area_cm2)),
```

```
    .groups = "drop"
  )
print(
  visceral_fat_area_descriptive,
  width = Inf,
  n = Inf
)
# 3. LINEAR MIXED-EFFECTS MODEL -----------------------------------------------
visceral_fat_area_model <- lmer(
  visceral_fat_area_cm2 ~
    group * experimental_period + (1 | id),
  data = visceral_fat_area_data,
  REML = TRUE,
  na.action = na.exclude
)
summary(visceral_fat_area_model)
# 4. GLOBAL FIXED-EFFECT TESTS -----------------------------------------------
visceral_fat_area_anova <- anova(
  visceral_fat_area_model,
  type = 3,
  ddf = "Satterthwaite"
)

visceral_fat_area_anova
# 5. ESTIMATED MARGINAL MEANS -----------------------------------------------
visceral_fat_area_emmeans <- emmeans(
  visceral_fat_area_model,
  ~ group * experimental_period
)
visceral_fat_area_emmeans
# 6. WITHIN-GROUP PRE–POST CONTRASTS ------------------------------------------
visceral_fat_area_period_contrasts <- contrast(
  visceral_fat_area_emmeans,
  method = "revpairwise",
  by = "group",
  adjust = "none"
)
summary(
  visceral_fat_area_period_contrasts,
  infer = c(TRUE, TRUE)
)
# 7. BETWEEN-GROUP CONTRASTS AT EACH PERIOD -----------------------------------
visceral_fat_area_group_contrasts <- contrast(
  visceral_fat_area_emmeans,
  method = "pairwise",
  by = "experimental_period",
```

```r
  adjust = "tukey"
)
summary(
  visceral_fat_area_group_contrasts,
  infer = c(TRUE, TRUE)
)
# 8. WITHIN-GROUP STANDARDIZED EFFECT SIZES -----------------------------------
visceral_fat_area_period_emmeans <- emmeans(
  visceral_fat_area_model,
  ~ experimental_period | group
)
visceral_fat_area_effect_sizes <- eff_size(
  visceral_fat_area_period_emmeans,
  sigma = sigma(visceral_fat_area_model),
  edf = df.residual(visceral_fat_area_model),
  method = "revpairwise"
)
visceral_fat_area_effect_sizes
# 9. ANALYTICAL SAMPLE -------------------------------------------------------
nobs(visceral_fat_area_model)
nlevels(
  droplevels(
    visceral_fat_area_data[
      !is.na(
        visceral_fat_area_data$visceral_fat_area_cm2
      ),
    ]
  )$id
)
################################################################################
#
# MODEL 2.1: CARDIORESPIRATORY FITNESS — VO2PEAK
################################################################################
#
# 1. DATA PREPARATION -------------------------------------------------------
vo2_peak_data <- cardio_data |>
  select(
    id,
    group,
    experimental_period,
    testing_time,
    vo2_peak_ml_kg_min
  ) |>
  mutate(
    id = factor(id),
    group = factor(
```

```
      group,
      levels = c("CG", "MT", "ET")
    ),
    experimental_period = factor(
      experimental_period,
      levels = c("pre", "post")
    ),
    testing_time = factor(
      testing_time,
      levels = c("morning", "evening")
    )
  )
stopifnot(
  is.numeric(vo2_peak_data$vo2_peak_ml_kg_min)
)

# 2. DESCRIPTIVE STATISTICS --------------------------------------------------
vo2_peak_descriptive <- vo2_peak_data |>
  group_by(
    group,
    experimental_period,
    testing_time
  ) |>
  summarise(
    n = sum(!is.na(vo2_peak_ml_kg_min)),
    mean = mean(
      vo2_peak_ml_kg_min,
      na.rm = TRUE
    ),
    standard_deviation = sd(
      vo2_peak_ml_kg_min,
      na.rm = TRUE
    ),
    median = median(
      vo2_peak_ml_kg_min,
      na.rm = TRUE
    ),
    missing_values = sum(
      is.na(vo2_peak_ml_kg_min)
    ),
    .groups = "drop"
  )
print(
  vo2_peak_descriptive,
  width = Inf,
  n = Inf
```

```
)
# 3. LINEAR MIXED-EFFECTS MODEL ----------------------------------------------
vo2_peak_model <- lmer(
  vo2_peak_ml_kg_min ~
    group * experimental_period * testing_time +
    (1 | id),
  data = vo2_peak_data,
  REML = TRUE,
  na.action = na.exclude
)
summary(vo2_peak_model)
# 4. GLOBAL FIXED-EFFECT TESTS ------------------------------------------------
vo2_peak_anova <- anova(
  vo2_peak_model,
  type = 3,
  ddf = "Satterthwaite"
)
vo2_peak_anova
# 5. ESTIMATED MARGINAL MEANS -------------------------------------------------
vo2_peak_emmeans <- emmeans(
  vo2_peak_model,
  ~ group * experimental_period * testing_time
)
vo2_peak_emmeans
# 6. PRE–POST CONTRASTS WITHIN EACH GROUP AND TESTING TIME -------------------
--
vo2_peak_period_contrasts <- contrast(
  vo2_peak_emmeans,
  method = "revpairwise",
  by = c(
    "group",
    "testing_time"
  ),
  adjust = "none"
)
summary(
  vo2_peak_period_contrasts,
  infer = c(TRUE, TRUE)
)
# 7. MORNING–EVENING CONTRASTS WITHIN EACH GROUP AND PERIOD --------------
-----
vo2_peak_time_contrasts <- contrast(
  vo2_peak_emmeans,
  method = "revpairwise",
  by = c(
    "group",
```

```
    "experimental_period"
  ),
  adjust = "none"
)
summary(
  vo2_peak_time_contrasts,
  infer = c(TRUE, TRUE)
)
# 8. BETWEEN-GROUP CONTRASTS AT EACH PERIOD AND TESTING TIME --------------
-----
vo2_peak_group_contrasts <- contrast(
  vo2_peak_emmeans,
  method = "pairwise",
  by = c(
    "experimental_period",
    "testing_time"
  ),
  adjust = "tukey"
)
summary(
  vo2_peak_group_contrasts,
  infer = c(TRUE, TRUE)
)
# 9. BASELINE BETWEEN-GROUP CONTRASTS BY TESTING TIME -------------------------
vo2_peak_baseline_emmeans <- emmeans(
  vo2_peak_model,
  ~ group | testing_time,
  at = list(
    experimental_period = "pre"
  )
)
vo2_peak_baseline_contrasts <- contrast(
  vo2_peak_baseline_emmeans,
  method = "pairwise",
  adjust = "tukey"
)
summary(
  vo2_peak_baseline_contrasts,
  infer = c(TRUE, TRUE)
)
# 10. STANDARDIZED PRE–POST EFFECT SIZES --------------------------------------
vo2_peak_period_emmeans <- emmeans(
  vo2_peak_model,
  ~ experimental_period |
    group * testing_time
```

```
)
vo2_peak_effect_sizes <- eff_size(
  vo2_peak_period_emmeans,
  sigma = sigma(vo2_peak_model),
  edf = df.residual(vo2_peak_model),
  method = "revpairwise"
)
vo2_peak_effect_sizes
# 11. DIRECT BETWEEN-GROUP COMPARISONS OF PRE–POST CHANGE ------------------
----
vo2_peak_change_emmeans <- emmeans(
  vo2_peak_model,
  ~ group * experimental_period
)
vo2_peak_change_contrasts <- contrast(
  vo2_peak_change_emmeans,
  interaction = c(
    "pairwise",
    "revpairwise"
  ),
  adjust = "tukey"
)

summary(
  vo2_peak_change_contrasts,
  infer = c(TRUE, TRUE)
)
# 12. ANALYTICAL SAMPLE -------------------------------------------------------
nobs(vo2_peak_model)
nlevels(
  droplevels(
    vo2_peak_data[
      !is.na(
        vo2_peak_data$vo2_peak_ml_kg_min
      ),
    ]
  )$id
)
################################################################################
#
# MODEL 2.2: MUSCULAR STRENGTH — BENCH PRESS 1RM
################################################################################
#
# 1. DATA PREPARATION ---------------------------------------------------------
bench_press_data <- strength_data |>
  filter(test_type == "bench_press") |>
```

```
  select(
    id,
    group,
    experimental_period,
    testing_time,
    one_rm_kg
  ) |>
  mutate(
    id = factor(id),
    group = factor(
      group,
      levels = c("CG", "MT", "ET")
    ),
    experimental_period = factor(
      experimental_period,
      levels = c("pre", "post")
    ),
    testing_time = factor(
      testing_time,
      levels = c("morning", "evening")
    )
  )
stopifnot(
  is.numeric(bench_press_data$one_rm_kg)
)
# 2. DESCRIPTIVE STATISTICS ----------------------------------------------------
bench_press_descriptive <- bench_press_data |>
  group_by(
    group,
    experimental_period,
    testing_time
  ) |>
  summarise(
    n = sum(!is.na(one_rm_kg)),
    mean = mean(one_rm_kg, na.rm = TRUE),
    standard_deviation = sd(one_rm_kg, na.rm = TRUE),
    median = median(one_rm_kg, na.rm = TRUE),
    missing_values = sum(is.na(one_rm_kg)),
    .groups = "drop"
  )
print(
  bench_press_descriptive,
  width = Inf,
  n = Inf
)
# 3. LINEAR MIXED-EFFECTS MODEL ------------------------------------------------
```

```
bench_press_model <- lmer(
  one_rm_kg ~
    group * experimental_period * testing_time +
    (1 | id),
  data = bench_press_data,
  REML = TRUE,
  na.action = na.exclude
)
summary(bench_press_model)
# 4. GLOBAL FIXED-EFFECT TESTS ------------------------------------------------
bench_press_anova <- anova(
  bench_press_model,
  type = 3,
  ddf = "Satterthwaite"
)
bench_press_anova
# 5. ESTIMATED MARGINAL MEANS ------------------------------------------------
bench_press_emmeans <- emmeans(
  bench_press_model,
  ~ group * experimental_period * testing_time
)
bench_press_emmeans
# 6. PRE–POST CONTRASTS WITHIN EACH GROUP AND TESTING TIME -------------------
--
bench_press_period_contrasts <- contrast(
  bench_press_emmeans,
  method = "revpairwise",
  by = c(
    "group",
    "testing_time"
  ),
  adjust = "none"
)
summary(
  bench_press_period_contrasts,
  infer = c(TRUE, TRUE)
)
# 7. MORNING–EVENING CONTRASTS WITHIN EACH GROUP AND PERIOD --------------
------
bench_press_time_contrasts <- contrast(
  bench_press_emmeans,
  method = "revpairwise",
  by = c(
    "group",
    "experimental_period"
  ),
```

```r
  adjust = "none"
)

summary(
  bench_press_time_contrasts,
  infer = c(TRUE, TRUE)
)
# 8. BETWEEN-GROUP CONTRASTS AT EACH PERIOD AND TESTING TIME --------------
-----
bench_press_group_contrasts <- contrast(
  bench_press_emmeans,
  method = "pairwise",
  by = c(
    "experimental_period",
    "testing_time"
  ),
  adjust = "tukey"
)
summary(
  bench_press_group_contrasts,
  infer = c(TRUE, TRUE)
)
# 9. BASELINE BETWEEN-GROUP CONTRASTS BY TESTING TIME --------------------------
bench_press_baseline_emmeans <- emmeans(
  bench_press_model,
  ~ group | testing_time,
  at = list(
    experimental_period = "pre"
  )
)
bench_press_baseline_contrasts <- contrast(
  bench_press_baseline_emmeans,
  method = "pairwise",
  adjust = "tukey"
)
summary(
  bench_press_baseline_contrasts,
  infer = c(TRUE, TRUE)
)
# 10. STANDARDIZED PRE–POST EFFECT SIZES --------------------------------------
bench_press_period_emmeans <- emmeans(
  bench_press_model,
  ~ experimental_period |
    group * testing_time
)
bench_press_effect_sizes <- eff_size(
```

```
  bench_press_period_emmeans,
  sigma = sigma(bench_press_model),
  edf = df.residual(bench_press_model),
  method = "revpairwise"
)
bench_press_effect_sizes
# 11. DIRECT BETWEEN-GROUP COMPARISONS OF PRE–POST CHANGE ------------------
----
bench_press_change_emmeans <- emmeans(
  bench_press_model,
  ~ group * experimental_period
)
bench_press_change_contrasts <- contrast(
  bench_press_change_emmeans,
  interaction = c(
    "pairwise",
    "revpairwise"
  ),
  adjust = "sidak"
)
summary(
  bench_press_change_contrasts,
  infer = c(TRUE, TRUE)
)
# 12. ANALYTICAL SAMPLE -------------------------------------------------------
nobs(bench_press_model)
nlevels(
  droplevels(
    bench_press_data[
      !is.na(
        bench_press_data$one_rm_kg
      ),
    ]
  )$id
)
################################################################################
#
# MODEL 2.3: MUSCULAR STRENGTH — LEG PRESS 1RM
################################################################################
#
# 1. DATA PREPARATION ---------------------------------------------------------
leg_press_data <- strength_data |>
  filter(test_type == "leg_press") |>
  select(
    id,
    group,
```

```r
    experimental_period,
    testing_time,
    one_rm_kg
  ) |>
  mutate(
    id = factor(id),
    group = factor(
      group,
      levels = c("CG", "MT", "ET")
    ),
    experimental_period = factor(
      experimental_period,
      levels = c("pre", "post")
    ),
    testing_time = factor(
      testing_time,
      levels = c("morning", "evening")
    )
  )
stopifnot(
  is.numeric(leg_press_data$one_rm_kg)
)
# 2. DESCRIPTIVE STATISTICS ---------------------------------------------------
leg_press_descriptive <- leg_press_data |>
  group_by(
    group,
    experimental_period,
    testing_time
  ) |>
  summarise(
    n = sum(!is.na(one_rm_kg)),
    mean = mean(one_rm_kg, na.rm = TRUE),
    standard_deviation = sd(one_rm_kg, na.rm = TRUE),
    median = median(one_rm_kg, na.rm = TRUE),
    missing_values = sum(is.na(one_rm_kg)),
    .groups = "drop"
  )
print(
  leg_press_descriptive,
  width = Inf,
  n = Inf
)
# 3. LINEAR MIXED-EFFECTS MODEL -----------------------------------------------
leg_press_model <- lmer(
  one_rm_kg ~
    group * experimental_period * testing_time +
```

```r
    (1 | id),
  data = leg_press_data,
  REML = TRUE,
  na.action = na.exclude
)
summary(leg_press_model)
# 4. GLOBAL FIXED-EFFECT TESTS ------------------------------------------------
leg_press_anova <- anova(
  leg_press_model,
  type = 3,
  ddf = "Satterthwaite"
)
leg_press_anova
# 5. ESTIMATED MARGINAL MEANS -------------------------------------------------
leg_press_emmeans <- emmeans(
  leg_press_model,
  ~ group * experimental_period * testing_time
)
leg_press_emmeans
# 6. PRE–POST CONTRASTS WITHIN EACH GROUP AND TESTING TIME -------------------
---
leg_press_period_contrasts <- contrast(
  leg_press_emmeans,
  method = "revpairwise",
  by = c("group", "testing_time"),
  adjust = "none"
)
summary(
  leg_press_period_contrasts,
  infer = c(TRUE, TRUE)
)
# 7. MORNING–EVENING CONTRASTS WITHIN EACH GROUP AND PERIOD --------------
-------
leg_press_time_contrasts <- contrast(
  leg_press_emmeans,
  method = "revpairwise",
  by = c("group", "experimental_period"),
  adjust = "none"
)

summary(
  leg_press_time_contrasts,
  infer = c(TRUE, TRUE)
)
# 8. BETWEEN-GROUP CONTRASTS AT EACH PERIOD AND TESTING TIME --------------
-----
```

```
leg_press_group_contrasts <- contrast(
  leg_press_emmeans,
  method = "pairwise",
  by = c("experimental_period", "testing_time"),
  adjust = "tukey"
)
summary(
  leg_press_group_contrasts,
  infer = c(TRUE, TRUE)
)
# 9. BASELINE BETWEEN-GROUP CONTRASTS BY TESTING TIME --------------------------
--
leg_press_baseline_emmeans <- emmeans(
  leg_press_model,
  ~ group | testing_time,
  at = list(
    experimental_period = "pre"
  )
)
leg_press_baseline_contrasts <- contrast(
  leg_press_baseline_emmeans,
  method = "pairwise",
  adjust = "tukey"
)

summary(
  leg_press_baseline_contrasts,
  infer = c(TRUE, TRUE)
)
# 10. STANDARDIZED PRE–POST EFFECT SIZES -----------------------------------------
leg_press_period_emmeans <- emmeans(
  leg_press_model,
  ~ experimental_period | group * testing_time
)
leg_press_effect_sizes <- eff_size(
  leg_press_period_emmeans,
  sigma = sigma(leg_press_model),
  edf = df.residual(leg_press_model),
  method = "revpairwise"
)
leg_press_effect_sizes
# 11. DIRECT BETWEEN-GROUP COMPARISONS OF PRE–POST CHANGE ------------------
----
leg_press_change_emmeans <- emmeans(
  leg_press_model,
  ~ group * experimental_period
```

```
)
leg_press_change_contrasts <- contrast(
  leg_press_change_emmeans,
  interaction = c("pairwise", "revpairwise"),
  adjust = "sidak"
)
summary(
  leg_press_change_contrasts,
  infer = c(TRUE, TRUE)
)
# 12. ANALYTICAL SAMPLE ----------------------------------------------------
nobs(leg_press_model)
nlevels(
  droplevels(
    leg_press_data[
      !is.na(leg_press_data$one_rm_kg),
    ]
  )$id
)
################################################################################
#
# MODEL 2.4: CARDIORESPIRATORY FITNESS — CPET DURATION
################################################################################
#
# 1. DATA PREPARATION ------------------------------------------------------
cpet_duration_data <- cardio_data |>
  select(
    id,
    group,
    experimental_period,
    testing_time,
    cpet_duration_min
  ) |>
  mutate(
    id = factor(id),
    group = factor(
      group,
      levels = c("CG", "MT", "ET")
    ),
    experimental_period = factor(
      experimental_period,
      levels = c("pre", "post")
    ),
    testing_time = factor(
      testing_time,
      levels = c("morning", "evening")
```

```
    )
  )
stopifnot(
  is.numeric(cpet_duration_data$cpet_duration_min)
)
# 2. DESCRIPTIVE STATISTICS ---------------------------------------------------
cpet_duration_descriptive <- cpet_duration_data |>
  group_by(
    group,
    experimental_period,
    testing_time
  ) |>
  summarise(
    n = sum(!is.na(cpet_duration_min)),
    mean = mean(cpet_duration_min, na.rm = TRUE),
    standard_deviation = sd(cpet_duration_min, na.rm = TRUE),
    median = median(cpet_duration_min, na.rm = TRUE),
    missing_values = sum(is.na(cpet_duration_min)),
    .groups = "drop"
  )

print(
  cpet_duration_descriptive,
  width = Inf,
  n = Inf
)
# 3. LINEAR MIXED-EFFECTS MODEL ----------------------------------------------
cpet_duration_model <- lmer(
  cpet_duration_min ~
    group * experimental_period * testing_time +
    (1 | id),
  data = cpet_duration_data,
  REML = TRUE,
  na.action = na.exclude
)
summary(cpet_duration_model)
# 4. GLOBAL FIXED-EFFECT TESTS ------------------------------------------------
cpet_duration_anova <- anova(
  cpet_duration_model,
  type = 3,
  ddf = "Satterthwaite"
)
cpet_duration_anova
# 5. ESTIMATED MARGINAL MEANS -------------------------------------------------
cpet_duration_emmeans <- emmeans(
  cpet_duration_model,
```

```
  ~ group * experimental_period * testing_time
)

cpet_duration_emmeans
# 6. PRE–POST CONTRASTS WITHIN EACH GROUP AND TESTING TIME -------------------
---
cpet_duration_period_contrasts <- contrast(
  cpet_duration_emmeans,
  method = "revpairwise",
  by = c("group", "testing_time"),
  adjust = "none"
)
summary(
  cpet_duration_period_contrasts,
  infer = c(TRUE, TRUE)
)
# 7. MORNING–EVENING CONTRASTS WITHIN EACH GROUP AND PERIOD --------------
-------
cpet_duration_time_contrasts <- contrast(
  cpet_duration_emmeans,
  method = "revpairwise",
  by = c("group", "experimental_period"),
  adjust = "none"
)
summary(
  cpet_duration_time_contrasts,
  infer = c(TRUE, TRUE)
)
# 8. BETWEEN-GROUP CONTRASTS AT EACH PERIOD AND TESTING TIME --------------
------
cpet_duration_group_contrasts <- contrast(
  cpet_duration_emmeans,
  method = "pairwise",
  by = c("experimental_period", "testing_time"),
  adjust = "tukey"
)
summary(
  cpet_duration_group_contrasts,
  infer = c(TRUE, TRUE)
)
# 9. BASELINE BETWEEN-GROUP CONTRASTS BY TESTING TIME --------------------------
cpet_duration_baseline_emmeans <- emmeans(
  cpet_duration_model,
  ~ group | testing_time,
  at = list(
    experimental_period = "pre"
```

```
  )
)
cpet_duration_baseline_contrasts <- contrast(
  cpet_duration_baseline_emmeans,
  method = "pairwise",
  adjust = "tukey"
)
summary(
  cpet_duration_baseline_contrasts,
  infer = c(TRUE, TRUE)
)
# 10. DIRECT BETWEEN-GROUP COMPARISONS OF PRE–POST CHANGE -----------------
------
cpet_duration_change_emmeans <- emmeans(
  cpet_duration_model,
  ~ group * experimental_period
)
cpet_duration_change_contrasts <- contrast(
  cpet_duration_change_emmeans,
  interaction = c("pairwise", "revpairwise"),
  adjust = "sidak"
)
summary(
  cpet_duration_change_contrasts,
  infer = c(TRUE, TRUE)
)
# 11. STANDARDIZED PRE–POST EFFECT SIZES --------------------------------------
cpet_duration_period_emmeans <- emmeans(
  cpet_duration_model,
  ~ experimental_period | group * testing_time
)
cpet_duration_effect_sizes <- eff_size(
  cpet_duration_period_emmeans,
  sigma = sigma(cpet_duration_model),
  edf = df.residual(cpet_duration_model),
  method = "revpairwise"
)
cpet_duration_effect_sizes
# 12. ANALYTICAL SAMPLE -----------------------------------------------------
nobs(cpet_duration_model)
nlevels(
  droplevels(
    cpet_duration_data[
      !is.na(cpet_duration_data$cpet_duration_min),
    ]
  )$id
```

```
)
################################################################################
#
# MODEL 2.5: CARDIORESPIRATORY FITNESS — MAXIMUM VELOCITY
################################################################################
#
# 1. DATA PREPARATION ----------------------------------------------------------
maximum_velocity_data <- cardio_data |>
  select(
    id,
    group,
    experimental_period,
    testing_time,
    maximum_velocity_kmh
  ) |>
  mutate(
    id = factor(id),
    group = factor(
      group,
      levels = c("CG", "MT", "ET")
    ),
    experimental_period = factor(
      experimental_period,
      levels = c("pre", "post")
    ),
    testing_time = factor(
      testing_time,
      levels = c("morning", "evening")
    )
  )
stopifnot(
  is.numeric(maximum_velocity_data$maximum_velocity_kmh)
)
# 2. DESCRIPTIVE STATISTICS ----------------------------------------------------
maximum_velocity_descriptive <- maximum_velocity_data |>
  group_by(
    group,
    experimental_period,
    testing_time
  ) |>
  summarise(
    n = sum(!is.na(maximum_velocity_kmh)),
    mean = mean(maximum_velocity_kmh, na.rm = TRUE),
    standard_deviation = sd(maximum_velocity_kmh, na.rm = TRUE),
    median = median(maximum_velocity_kmh, na.rm = TRUE),
    missing_values = sum(is.na(maximum_velocity_kmh)),
```

```r
    .groups = "drop"
  )
print(
  maximum_velocity_descriptive,
  width = Inf,
  n = Inf
)
# 3. LINEAR MIXED-EFFECTS MODEL ------------------------------------------------
maximum_velocity_model <- lmer(
  maximum_velocity_kmh ~
    group * experimental_period * testing_time +
    (1 | id),
  data = maximum_velocity_data,
  REML = TRUE,
  na.action = na.exclude
)
summary(maximum_velocity_model)
# 4. GLOBAL FIXED-EFFECT TESTS -------------------------------------------------
maximum_velocity_anova <- anova(
  maximum_velocity_model,
  type = 3,
  ddf = "Satterthwaite"
)
maximum_velocity_anova
# 5. ESTIMATED MARGINAL MEANS --------------------------------------------------
maximum_velocity_emmeans <- emmeans(
  maximum_velocity_model,
  ~ group * experimental_period * testing_time
)
maximum_velocity_emmeans
# 6. PRE–POST CONTRASTS WITHIN EACH GROUP AND TESTING TIME ---------------------
maximum_velocity_period_contrasts <- contrast(
  maximum_velocity_emmeans,
  method = "revpairwise",
  by = c("group", "testing_time"),
  adjust = "none"
)
summary(
  maximum_velocity_period_contrasts,
  infer = c(TRUE, TRUE)
)
# 7. MORNING–EVENING CONTRASTS WITHIN EACH GROUP AND PERIOD ---------------------
maximum_velocity_time_contrasts <- contrast(
  maximum_velocity_emmeans,
```

```
  method = "revpairwise",
  by = c("group", "experimental_period"),
  adjust = "none"
)
summary(
  maximum_velocity_time_contrasts,
  infer = c(TRUE, TRUE)
)
# 8. BETWEEN-GROUP CONTRASTS AT EACH PERIOD AND TESTING TIME --------------
------
maximum_velocity_group_contrasts <- contrast(
  maximum_velocity_emmeans,
  method = "pairwise",
  by = c("experimental_period", "testing_time"),
  adjust = "tukey"
)
summary(
  maximum_velocity_group_contrasts,
  infer = c(TRUE, TRUE)
)
# 9. BASELINE BETWEEN-GROUP CONTRASTS BY TESTING TIME --------------------------
maximum_velocity_baseline_emmeans <- emmeans(
  maximum_velocity_model,
  ~ group | testing_time,
  at = list(
    experimental_period = "pre"
  )
)
maximum_velocity_baseline_contrasts <- contrast(
  maximum_velocity_baseline_emmeans,
  method = "pairwise",
  adjust = "tukey"
)
summary(
  maximum_velocity_baseline_contrasts,
  infer = c(TRUE, TRUE)
)
# 10. DIRECT BETWEEN-GROUP COMPARISONS OF PRE–POST CHANGE ------------------
------
maximum_velocity_change_emmeans <- emmeans(
  maximum_velocity_model,
  ~ group * experimental_period
)
maximum_velocity_change_contrasts <- contrast(
  maximum_velocity_change_emmeans,
  interaction = c("pairwise", "revpairwise"),
```

```r
  adjust = "sidak"
)
summary(
  maximum_velocity_change_contrasts,
  infer = c(TRUE, TRUE)
)

# 11. STANDARDIZED PRE–POST EFFECT SIZES ----------------------------------------
maximum_velocity_period_emmeans <- emmeans(
  maximum_velocity_model,
  ~ experimental_period | group * testing_time
)
maximum_velocity_effect_sizes <- eff_size(
  maximum_velocity_period_emmeans,
  sigma = sigma(maximum_velocity_model),
  edf = df.residual(maximum_velocity_model),
  method = "revpairwise"
)
maximum_velocity_effect_sizes
# 12. ANALYTICAL SAMPLE -------------------------------------------------------
nobs(maximum_velocity_model)
nlevels(
  droplevels(
    maximum_velocity_data[
      !is.na(maximum_velocity_data$maximum_velocity_kmh),
    ]
  )$id
)
################################################################################
#
# MODEL 3.1: HEART RATE
################################################################################
#


# 1. DATA PREPARATION ---------------------------------------------------------

heart_rate_data <- cardio_data |>
  select(
    id,
    group,
    experimental_period,
    testing_time,
    measurement_moment,
    heart_rate
  ) |>
```

```r
  mutate(
    id = factor(id),
    group = factor(
      group,
      levels = c("CG", "MT", "ET")
    ),
    experimental_period = factor(
      experimental_period,
      levels = c("pre", "post")
    ),
    testing_time = factor(
      testing_time,
      levels = c("morning", "evening")
    ),
    measurement_moment = factor(
      measurement_moment,
      levels = c("rest", "post_test")
    )
  )

stopifnot(
  is.numeric(heart_rate_data$heart_rate)
)
# 2. DESCRIPTIVE STATISTICS ---------------------------------------------------
heart_rate_descriptive <- heart_rate_data |>
  group_by(
    group,
    experimental_period,
    testing_time,
    measurement_moment
  ) |>
  summarise(
    n = sum(!is.na(heart_rate)),
    mean = mean(heart_rate, na.rm = TRUE),
    standard_deviation = sd(heart_rate, na.rm = TRUE),
    median = median(heart_rate, na.rm = TRUE),
    missing_values = sum(is.na(heart_rate)),
    .groups = "drop"
  )
print(
  heart_rate_descriptive,
  width = Inf,
  n = Inf
)
# 3. LINEAR MIXED-EFFECTS MODEL ------------------------------------------------
heart_rate_model <- lmer(
```

```
  heart_rate ~
    group *
    experimental_period *
    testing_time *
    measurement_moment +
    (1 | id),
  data = heart_rate_data,
  REML = TRUE,
  na.action = na.exclude
)
summary(heart_rate_model)
# 4. GLOBAL FIXED-EFFECT TESTS -------------------------------------------------
heart_rate_anova <- anova(
  heart_rate_model,
  type = 3,
  ddf = "Satterthwaite"
)
heart_rate_anova
# 5. ESTIMATED MARGINAL MEANS --------------------------------------------------
heart_rate_emmeans <- emmeans(
  heart_rate_model,
  ~ group *
    experimental_period *
    testing_time *
    measurement_moment
)
heart_rate_emmeans
# 6. PRE–POST CONTRASTS WITHIN GROUP, TIME, AND MEASUREMENT MOMENT --
------------
heart_rate_period_contrasts <- contrast(
  heart_rate_emmeans,
  method = "revpairwise",
  by = c(
    "group",
    "testing_time",
    "measurement_moment"
  ),
  adjust = "none"
)
summary(
  heart_rate_period_contrasts,
  infer = c(TRUE, TRUE)
)
# 7. REST–POST-TEST CONTRASTS --------------------------------------------------
heart_rate_moment_contrasts <- contrast(
  heart_rate_emmeans,
```

```r
  method = "revpairwise",
  by = c(
    "group",
    "experimental_period",
    "testing_time"
  ),
  adjust = "none"
)
summary(
  heart_rate_moment_contrasts,
  infer = c(TRUE, TRUE)
)
# 8. MORNING–EVENING CONTRASTS ------------------------------------------------
heart_rate_time_contrasts <- contrast(
  heart_rate_emmeans,
  method = "revpairwise",
  by = c(
    "group",
    "experimental_period",
    "measurement_moment"
  ),
  adjust = "none"
)
summary(
  heart_rate_time_contrasts,
  infer = c(TRUE, TRUE)
)
# 9. BETWEEN-GROUP CONTRASTS -------------------------------------------------
heart_rate_group_contrasts <- contrast(
  heart_rate_emmeans,
  method = "pairwise",
  by = c(
    "experimental_period",
    "testing_time",
    "measurement_moment"
  ),
  adjust = "tukey"
)
summary(
  heart_rate_group_contrasts,
  infer = c(TRUE, TRUE)
)
# 10. DIRECT GROUP × EXPERIMENTAL PERIOD CONTRASTS ----------------------------

heart_rate_change_emmeans <- emmeans(
  heart_rate_model,
```

```
  ~ group * experimental_period
)
heart_rate_change_contrasts <- contrast(
  heart_rate_change_emmeans,
  interaction = c("pairwise", "revpairwise"),
  adjust = "sidak"
)
summary(
  heart_rate_change_contrasts,
  infer = c(TRUE, TRUE)
)
# 11. DIRECT GROUP × PERIOD × MEASUREMENT MOMENT CONTRASTS ----------------
------
heart_rate_change_by_moment_emmeans <- emmeans(
  heart_rate_model,
  ~ group * experimental_period | measurement_moment
)
heart_rate_change_by_moment_contrasts <- contrast(
  heart_rate_change_by_moment_emmeans,
  interaction = c("pairwise", "revpairwise"),
  adjust = "sidak"
)
summary(
  heart_rate_change_by_moment_contrasts,
  infer = c(TRUE, TRUE)
)
# 12. STANDARDIZED PRE–POST EFFECT SIZES ---------------------------------------
heart_rate_period_emmeans <- emmeans(
  heart_rate_model,
  ~ experimental_period |
    group *
    testing_time *
    measurement_moment
)
heart_rate_effect_sizes <- eff_size(
  heart_rate_period_emmeans,
  sigma = sigma(heart_rate_model),
  edf = df.residual(heart_rate_model),
  method = "revpairwise"
)
heart_rate_effect_sizes
# 13. ANALYTICAL SAMPLE --------------------------------------------------------
nobs(heart_rate_model)
nlevels(
  droplevels(
    heart_rate_data[
```

```
      !is.na(heart_rate_data$heart_rate),
    ]
  )$id
)
################################################################################
#
# MODEL 3.2: SYSTOLIC BLOOD PRESSURE
################################################################################
#


# 1. DATA PREPARATION --------------------------------------------------------
systolic_blood_pressure_data <- cardio_data |>
  select(
    id,
    group,
    experimental_period,
    testing_time,
    measurement_moment,
    systolic_blood_pressure
  ) |>
  mutate(
    id = factor(id),
    group = factor(
      group,
      levels = c("CG", "MT", "ET")
    ),
    experimental_period = factor(
      experimental_period,
      levels = c("pre", "post")
    ),
    testing_time = factor(
      testing_time,
      levels = c("morning", "evening")
    ),
    measurement_moment = factor(
      measurement_moment,
      levels = c("rest", "post_test")
    )
  )
stopifnot(
  is.numeric(
    systolic_blood_pressure_data$systolic_blood_pressure
  )
)
# 2. DESCRIPTIVE STATISTICS --------------------------------------------------
```

```
systolic_blood_pressure_descriptive <- systolic_blood_pressure_data |>
  group_by(
    group,
    experimental_period,
    testing_time,
    measurement_moment
  ) |>
  summarise(
    n = sum(!is.na(systolic_blood_pressure)),
    mean = mean(
      systolic_blood_pressure,
      na.rm = TRUE
    ),
    standard_deviation = sd(
      systolic_blood_pressure,
      na.rm = TRUE
    ),
    median = median(
      systolic_blood_pressure,
      na.rm = TRUE
    ),
    missing_values = sum(
      is.na(systolic_blood_pressure)
    ),
    .groups = "drop"
  )
print(
  systolic_blood_pressure_descriptive,
  width = Inf,
  n = Inf
)
# 3. LINEAR MIXED-EFFECTS MODEL ------------------------------------------------
systolic_blood_pressure_model <- lmer(
  systolic_blood_pressure ~
    group *
    experimental_period *
    testing_time *
    measurement_moment +
    (1 | id),
  data = systolic_blood_pressure_data,
  REML = TRUE,
  na.action = na.exclude
)
summary(systolic_blood_pressure_model)
# 4. GLOBAL FIXED-EFFECT TESTS -------------------------------------------------
systolic_blood_pressure_anova <- anova(
```

```
  systolic_blood_pressure_model,
  type = 3,
  ddf = "Satterthwaite"
)

systolic_blood_pressure_anova
# 5. ESTIMATED MARGINAL MEANS -----------------------------------------------
systolic_blood_pressure_emmeans <- emmeans(
  systolic_blood_pressure_model,
  ~ group *
    experimental_period *
    testing_time *
    measurement_moment
)
systolic_blood_pressure_emmeans
# 6. PRE–POST CONTRASTS -----------------------------------------------------
systolic_blood_pressure_period_contrasts <- contrast(
  systolic_blood_pressure_emmeans,
  method = "revpairwise",
  by = c(
    "group",
    "testing_time",
    "measurement_moment"
  ),
  adjust = "none"
)
summary(
  systolic_blood_pressure_period_contrasts,
  infer = c(TRUE, TRUE)
)
# 7. REST–POST-TEST CONTRASTS -----------------------------------------------
systolic_blood_pressure_moment_contrasts <- contrast(
  systolic_blood_pressure_emmeans,
  method = "revpairwise",
  by = c(
    "group",
    "experimental_period",
    "testing_time"
  ),
  adjust = "none"
)
summary(
  systolic_blood_pressure_moment_contrasts,
  infer = c(TRUE, TRUE)
)
# 8. MORNING–EVENING CONTRASTS ----------------------------------------------
```

```r
systolic_blood_pressure_time_contrasts <- contrast(
  systolic_blood_pressure_emmeans,
  method = "revpairwise",
  by = c(
    "group",
    "experimental_period",
    "measurement_moment"
  ),
  adjust = "none"
)
summary(
  systolic_blood_pressure_time_contrasts,
  infer = c(TRUE, TRUE)
)
# 9. BETWEEN-GROUP CONTRASTS -------------------------------------------------------
systolic_blood_pressure_group_contrasts <- contrast(
  systolic_blood_pressure_emmeans,
  method = "pairwise",
  by = c(
    "experimental_period",
    "testing_time",
    "measurement_moment"
  ),
  adjust = "tukey"
)
summary(
  systolic_blood_pressure_group_contrasts,
  infer = c(TRUE, TRUE)
)
# 10. DIRECT GROUP × EXPERIMENTAL PERIOD CONTRASTS ----------------------------
systolic_blood_pressure_change_emmeans <- emmeans(
  systolic_blood_pressure_model,
  ~ group * experimental_period
)
systolic_blood_pressure_change_contrasts <- contrast(
  systolic_blood_pressure_change_emmeans,
  interaction = c(
    "pairwise",
    "revpairwise"
  ),
  adjust = "sidak"
)
summary(
  systolic_blood_pressure_change_contrasts,
  infer = c(TRUE, TRUE)
)
```

```
# 11. DIRECT GROUP × PERIOD CONTRASTS BY MEASUREMENT MOMENT -------------
-------
systolic_blood_pressure_change_by_moment_emmeans <- emmeans(
  systolic_blood_pressure_model,
  ~ group * experimental_period |
    measurement_moment
)
systolic_blood_pressure_change_by_moment_contrasts <- contrast(
  systolic_blood_pressure_change_by_moment_emmeans,
  interaction = c(
    "pairwise",
    "revpairwise"
  ),
  adjust = "sidak"
)
summary(
  systolic_blood_pressure_change_by_moment_contrasts,
  infer = c(TRUE, TRUE)
)
# 12. STANDARDIZED PRE–POST EFFECT SIZES --------------------------------------
systolic_blood_pressure_period_emmeans <- emmeans(
  systolic_blood_pressure_model,
  ~ experimental_period |
    group *
    testing_time *
    measurement_moment
)
systolic_blood_pressure_effect_sizes <- eff_size(
  systolic_blood_pressure_period_emmeans,
  sigma = sigma(systolic_blood_pressure_model),
  edf = df.residual(systolic_blood_pressure_model),
  method = "revpairwise"
)
systolic_blood_pressure_effect_sizes
# 13. ANALYTICAL SAMPLE ------------------------------------------------------
nobs(systolic_blood_pressure_model)
nlevels(
  droplevels(
    systolic_blood_pressure_data[
      !is.na(
        systolic_blood_pressure_data$systolic_blood_pressure
      ),
    ]
  )$id
)
```

```r
################################################################################
#
# MODEL 3.3: DIASTOLIC BLOOD PRESSURE
################################################################################
#
# 1. DATA PREPARATION ----------------------------------------------------------
diastolic_blood_pressure_data <- cardio_data |>
  select(
    id,
    group,
    experimental_period,
    testing_time,
    measurement_moment,
    diastolic_blood_pressure
  ) |>
  mutate(
    id = factor(id),
    group = factor(
      group,
      levels = c("CG", "MT", "ET")
    ),
    experimental_period = factor(
      experimental_period,
      levels = c("pre", "post")
    ),
    testing_time = factor(
      testing_time,
      levels = c("morning", "evening")
    ),
    measurement_moment = factor(
      measurement_moment,
      levels = c("rest", "post_test")
    )
  )
stopifnot(
  is.numeric(
    diastolic_blood_pressure_data$diastolic_blood_pressure
  )
)
# 2. DESCRIPTIVE STATISTICS ----------------------------------------------------
diastolic_blood_pressure_descriptive <- diastolic_blood_pressure_data |>
  group_by(
    group,
    experimental_period,
    testing_time,
    measurement_moment
```

```
  ) |>
  summarise(
    n = sum(!is.na(diastolic_blood_pressure)),
    mean = mean(
      diastolic_blood_pressure,
      na.rm = TRUE
    ),
    standard_deviation = sd(
      diastolic_blood_pressure,
      na.rm = TRUE
    ),
    median = median(
      diastolic_blood_pressure,
      na.rm = TRUE
    ),
    missing_values = sum(
      is.na(diastolic_blood_pressure)
    ),
    .groups = "drop"
  )
print(
  diastolic_blood_pressure_descriptive,
  width = Inf,
  n = Inf
)
# 3. LINEAR MIXED-EFFECTS MODEL ------------------------------------------------
diastolic_blood_pressure_model <- lmer(
  diastolic_blood_pressure ~
    group *
    experimental_period *
    testing_time *
    measurement_moment +
    (1 | id),
  data = diastolic_blood_pressure_data,
  REML = TRUE,
  na.action = na.exclude
)
summary(diastolic_blood_pressure_model)
# 4. GLOBAL FIXED-EFFECT TESTS -------------------------------------------------
diastolic_blood_pressure_anova <- anova(
  diastolic_blood_pressure_model,
  type = 3,
  ddf = "Satterthwaite"
)
diastolic_blood_pressure_anova
# 5. ESTIMATED MARGINAL MEANS --------------------------------------------------
```

```
diastolic_blood_pressure_emmeans <- emmeans(
  diastolic_blood_pressure_model,
  ~ group *
    experimental_period *
    testing_time *
    measurement_moment
)
diastolic_blood_pressure_emmeans
# 6. PRE–POST CONTRASTS ----------------------------------------------------------
diastolic_blood_pressure_period_contrasts <- contrast(
  diastolic_blood_pressure_emmeans,
  method = "revpairwise",
  by = c(
    "group",
    "testing_time",
    "measurement_moment"
  ),
  adjust = "none"
)
summary(
  diastolic_blood_pressure_period_contrasts,
  infer = c(TRUE, TRUE)
)
# 7. REST–POST-TEST CONTRASTS ----------------------------------------------------
diastolic_blood_pressure_moment_contrasts <- contrast(
  diastolic_blood_pressure_emmeans,
  method = "revpairwise",
  by = c(
    "group",
    "experimental_period",
    "testing_time"
  ),
  adjust = "none"
)
summary(
  diastolic_blood_pressure_moment_contrasts,
  infer = c(TRUE, TRUE)
)
# 8. MORNING–EVENING CONTRASTS -------------------------------------------------
diastolic_blood_pressure_time_contrasts <- contrast(
  diastolic_blood_pressure_emmeans,
  method = "revpairwise",
  by = c(
    "group",
    "experimental_period",
    "measurement_moment"
```

```
  ),
  adjust = "none"
)
summary(
  diastolic_blood_pressure_time_contrasts,
  infer = c(TRUE, TRUE)
)
# 9. BETWEEN-GROUP CONTRASTS ---------------------------------------------------
diastolic_blood_pressure_group_contrasts <- contrast(
  diastolic_blood_pressure_emmeans,
  method = "pairwise",
  by = c(
    "experimental_period",
    "testing_time",
    "measurement_moment"
  ),
  adjust = "tukey"
)
summary(
  diastolic_blood_pressure_group_contrasts,
  infer = c(TRUE, TRUE)
)
# 10. DIRECT GROUP × EXPERIMENTAL PERIOD CONTRASTS -----------------------------
diastolic_blood_pressure_change_emmeans <- emmeans(
  diastolic_blood_pressure_model,
  ~ group * experimental_period
)
diastolic_blood_pressure_change_contrasts <- contrast(
  diastolic_blood_pressure_change_emmeans,
  interaction = c(
    "pairwise",
    "revpairwise"
  ),
  adjust = "sidak"
)
summary(
  diastolic_blood_pressure_change_contrasts,
  infer = c(TRUE, TRUE)
)
# 11. DIRECT GROUP × PERIOD CONTRASTS BY MEASUREMENT MOMENT -------------
-------
diastolic_blood_pressure_change_by_moment_emmeans <- emmeans(
  diastolic_blood_pressure_model,
  ~ group * experimental_period |
    measurement_moment
)
```

```r
diastolic_blood_pressure_change_by_moment_contrasts <- contrast(
  diastolic_blood_pressure_change_by_moment_emmeans,
  interaction = c(
    "pairwise",
    "revpairwise"
  ),
  adjust = "sidak"
)
summary(
  diastolic_blood_pressure_change_by_moment_contrasts,
  infer = c(TRUE, TRUE)
)
# 12. STANDARDIZED PRE–POST EFFECT SIZES ---------------------------------------
diastolic_blood_pressure_period_emmeans <- emmeans(
  diastolic_blood_pressure_model,
  ~ experimental_period |
    group *
    testing_time *
    measurement_moment
)
diastolic_blood_pressure_effect_sizes <- eff_size(
  diastolic_blood_pressure_period_emmeans,
  sigma = sigma(diastolic_blood_pressure_model),
  edf = df.residual(diastolic_blood_pressure_model),
  method = "revpairwise"
)
diastolic_blood_pressure_effect_sizes
# 13. ANALYTICAL SAMPLE -------------------------------------------------------
nobs(diastolic_blood_pressure_model)
nlevels(
  droplevels(
    diastolic_blood_pressure_data[
      !is.na(
        diastolic_blood_pressure_data$diastolic_blood_pressure
      ),
    ]
  )$id
)
```
